# Supplementary material for: A Novel Chemically Differentiated Mouse Embryonic Stem Cell-Based Model to Study Liver Stages of Plasmodium berghei
Source: Stem Cell Reports. 2020 May 21;14(6):1123–34. doi: 10.1016/j.stemcr.2020.04.010 (PMC7355138; doi:10.1016/j.stemcr.2020.04.010)
Supplement: Document S1. Supplemental Experimental Procedures and Figures S1–S5 [file mmc1.pdf]

Stem Cell Reports, Volume *14*

Supplemental Information

A Novel Chemically Differentiated Mouse Embryonic Stem Cell-Based Model to Study Liver Stages of *Plasmodium berghei*

Jaishree Tripathi, Charis-Patricia Segeritz, Gareth Griffiths, Wendy Bushell, Ludovic Vallier, William C. Skarnes, Maria M. Mota, and Oliver Billker

A novel chemically differentiated mouse embryonic stem cell based model to study liver stages of *Plasmodium berghei*

Jaishree Tripathi[1], Charis-Patricia Segeritz[2], Gareth Griffiths[1], Wendy Bushell[1], Ludovic Vallier[1,2], William C. Skarnes[3], Maria M. Mota[4], Oliver Billker[1, 5*]

[1] *Wellcome Trust Sanger Institute, Wellcome Genome Campus, Hinxton, Cambridge, UK*

[2] *Wellcome Trust and Medical Research Council Stem Cell Institute, Department of Surgery, University of Cambridge, Cambridge, UK*

[3] *The Jackson Laboratory for Genomic Medicine, Ten Discovery Drive, Farmington, CT 06032*

[4] *Unidade de Malária, Instituto de Medicina Molecular, Universidade de Lisboa, Lisboa, Portugal*

[5] *Molecular Infection Medicine Sweden and Molecular Biology Department, Umeå University, 90187 Umeå, Sweden*

**Correspondence: oliver.billker@umu.se*

Supplementary Figures:

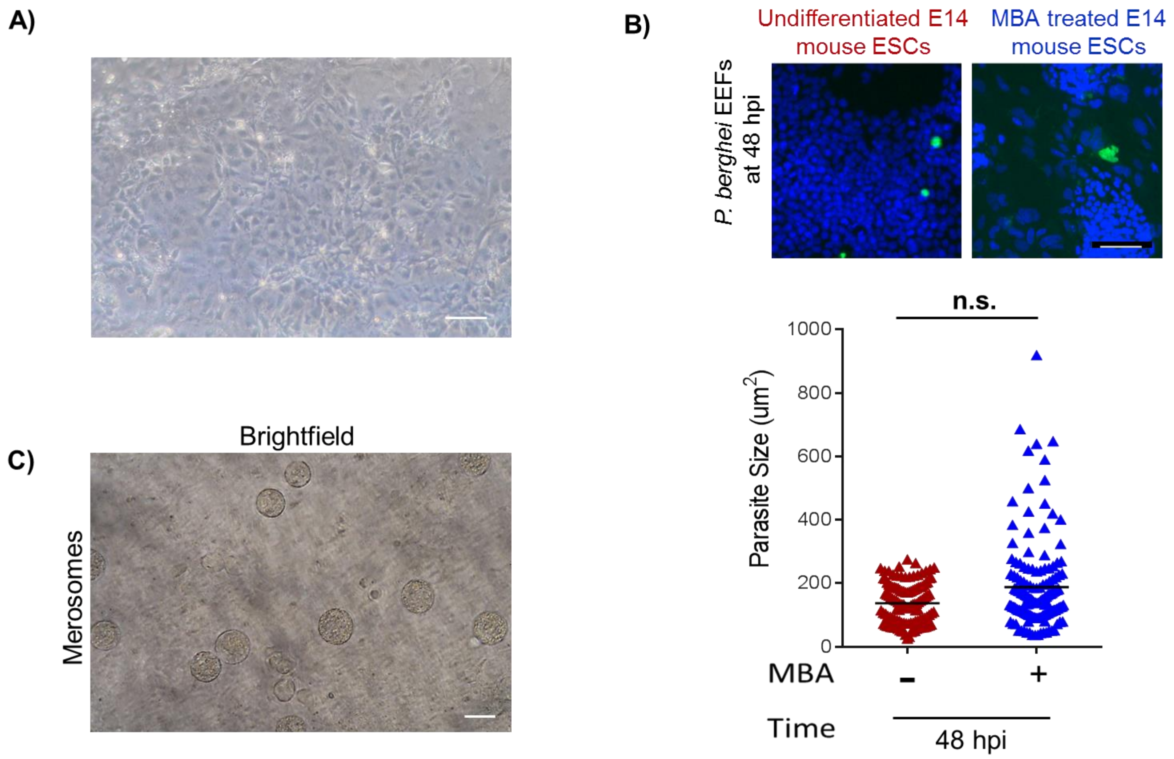

D)

Cell Type	Mice Injected with Merosomes/Supernatant	Mice Infected
MBA diff E14	5	5
Undiff E14	5	0

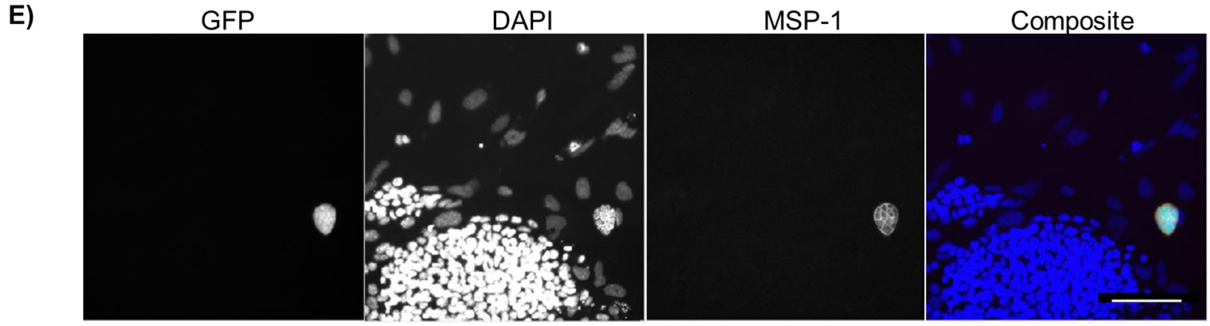

Fig S1: MBA-differentiated E14 mouse ESCs support complete *P. berghei* LS development. a) A bright field image showing morphology of MBA-treated E14 mouse ESCs on day 3 of differentiation. b) A widefield fluorescence image showing infected undifferentiated and MBA differentiated E14 mouse ESCs stained with DAPI (blue) and anti-GFP-Alexa-488 antibody (green) to visualize host cell nuclei and EEFs respectively at 48 hpi. Scale bar = 100 µm. Size of EEFs fixed at 48 hpi was quantified in undifferentiated (red) and MBA differentiated (blue) E14 mouse ESCs through automated

microscopy. Student's t- test was performed on mean parasite size from three independent experiments. Key: n. s. = not significant. c) A bright field image of merosomes released from infected MBA differentiated E14 mouse ESCs. Scale bar = 50 µm. d) Two and three TO mice were injected per condition with cell culture supernatant from infected wells from two independent experiments respectively. e) Infected MBA differentiated E14 mouse ESCs were fixed, permeabilized and stained with mouse anti-MSP-1 primary antibody followed by anti-mouse-Alexa Fluor-555 secondary antibody (red) to visualize MSP-1 expression at 65 hpi. DAPI (blue) and anti-GFP-Alexa Fluor-488 antibody (green) staining was performed on the same culture to visualize nuclei and EEFs respectively. Scale bar = 100 µm.

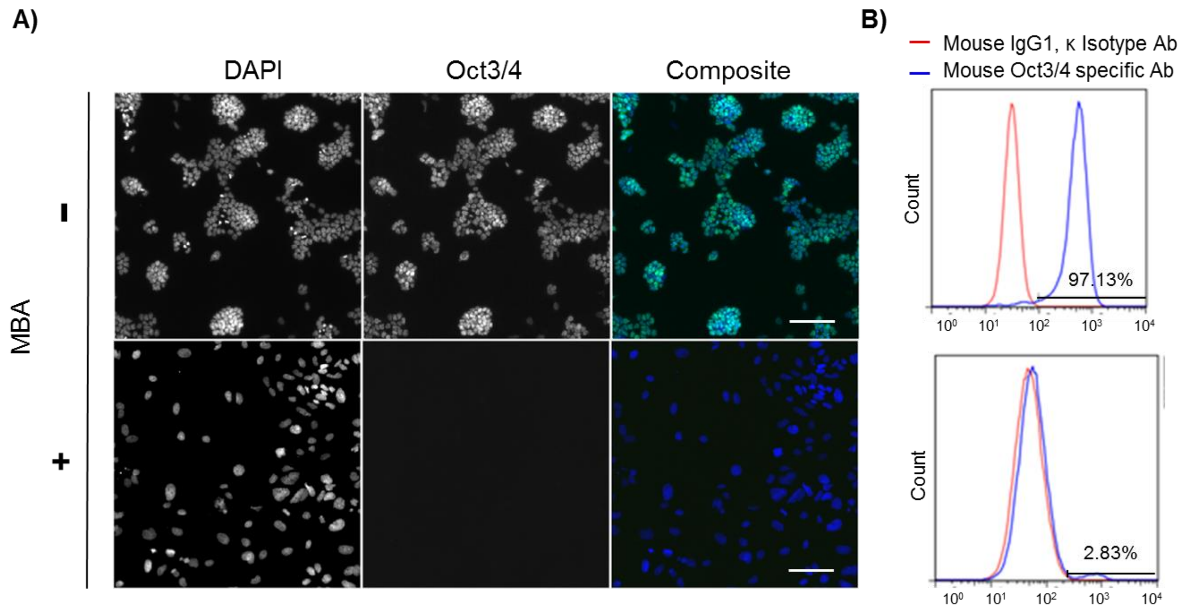

Fig S2: Loss of pluripotent marker expression after MBA treatment. Undifferentiated and MBA-differentiated E14 mouse ESCs were fixed, permeabilized and stained with a) DAPI and mouse anti-Oct3/4 primary antibody followed by donkey anti-mouse-Alexa 488 secondary antibody to visualise host cell nuclei and Oct3/4 transcription factors respectively. The imaging was done using wide field fluorescence microscopy. Scale bar = 100 µm. b) Mouse IgG1 isotype control (red) or mouse Oct3/4 specific antibody (blue) (BD Stemflow Human and Mouse Pluripotent Stem Cell Analysis Kit) to quantify undifferentiated (Oct3/4 positive) population by flow cytometry.

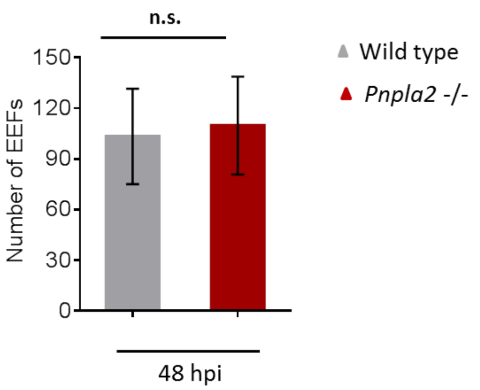

Fig S3: Number of EEFs in WT and Pnpla2 KO E14 cells. Number of EEFs in triplicate wells for three independent experiments. Error bar represents mean ± standard error of means (SEM). Key: n.s = not significant.

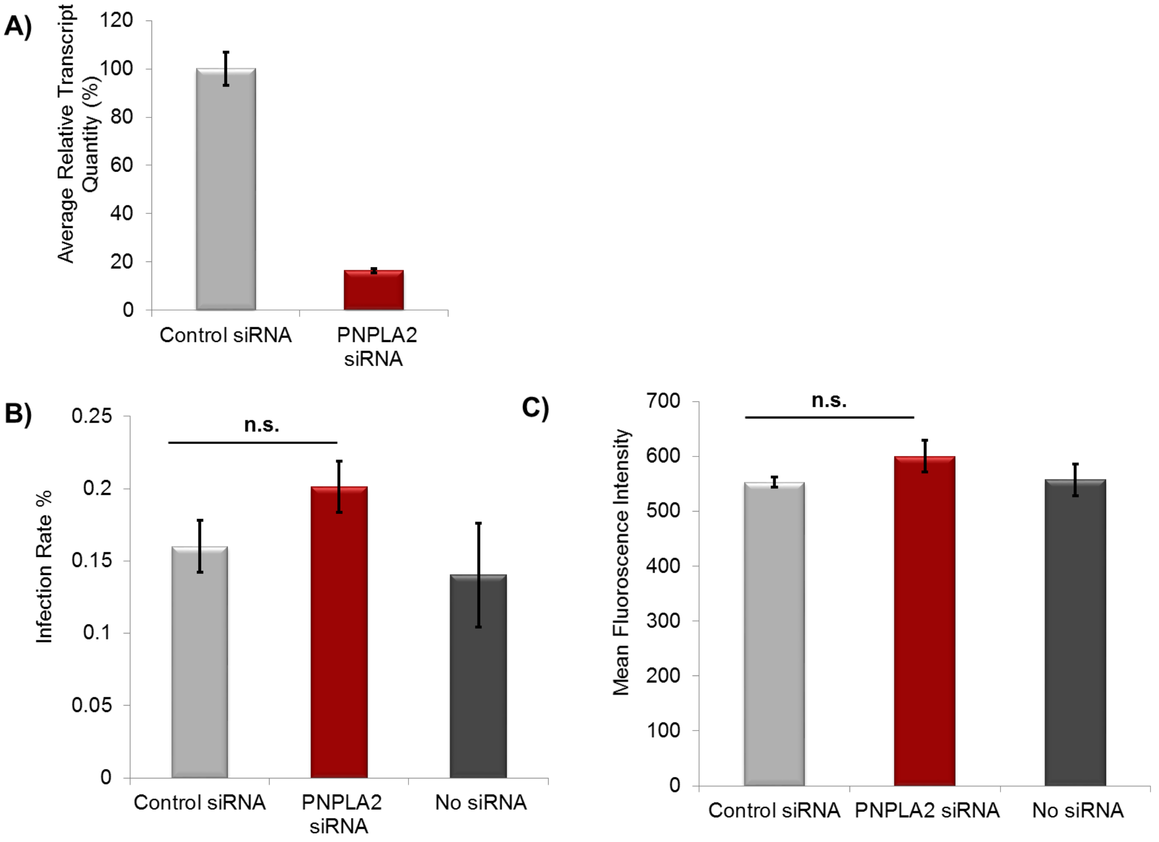

Fig S4: *PNPLA2* knockdown in healthy dermal fibroblast does not affect *P. berghei* infection and growth. a) *PNPLA2* transcript level was measured in control and *PNPLA2* siRNA transfected healthy fibroblasts by qRTPCR 48 hours after reverse transfection. Error bars represent mean ± standard deviation of duplicates from a representative experiment out of two independent biological replicates. b) Infection rate, and, c) MFI were measured in infected, control, *PNPLA2* and no siRNA transfected healthy fibroblasts at 48 hours post infection (hpi) by flow cytometry. Error bars represent mean ± standard deviation of triplicates from a representative experiment. Student's t test was

performed on mean infection rate (b) and MFI (c) from three independent experiments. n.s.= not significant.

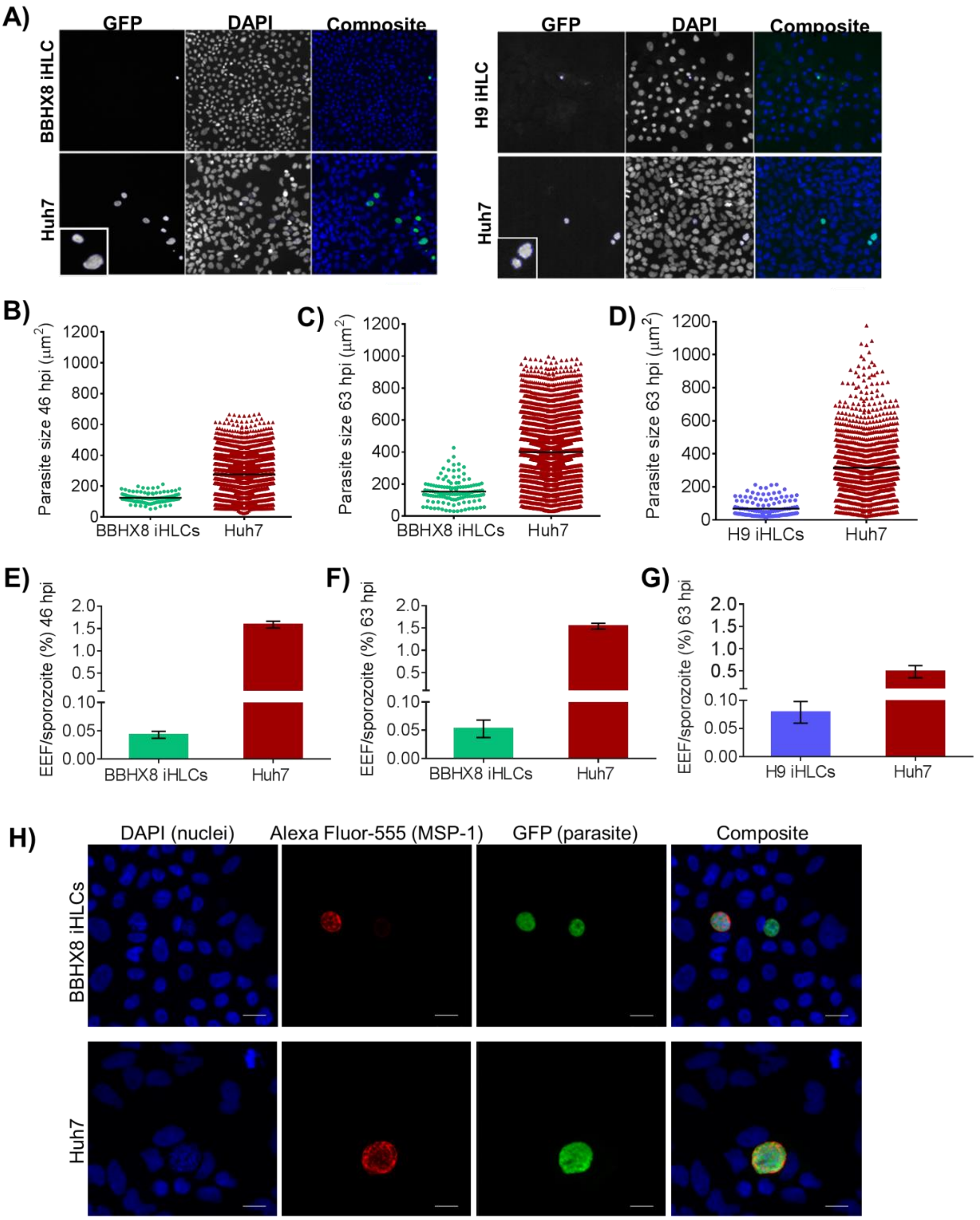

I)

Cell Type	Mice Injected with Merosomes	Mice Infected
BBHX8 iHLCs	4	0
Huh7 cells	4	4

Fig S5: *P. berghei* LS infection in BBHX8 and H9 iHLCs. a) Infected BBHX8 iHLCs, H9 iHLCs and Huh7 cells were fixed and stained with DAPI to visualize the nuclei (blue) and an anti-GFP-Alexa Fluor-488 antibody (green) to visualize the EEFs at 63 hpi. The EEFs were imaged automatically using the Spot Detector Algorithm. The objects detected by the algorithm are either accepted (denoted by blue ring) or rejected (denoted by orange ring) based on pre-set object selection parameters. b), c) and d) show parasite sizes in BBHX8 and H9 iHLCs at mid and late LS time points. Data shown here is from a representative experiment out of two independent biological replicates. e), f) and g) show sporozoite to EEF conversion (%) for BBHX8 and H9 iHLCs compared to their respective Huh7 controls. Data represents mean ± standard deviation of triplicates from a representative experiment out of two independent biological replicates. h) Infected BBHX8 iHLCs and Huh7 cells were fixed, permeabilized and stained with DAPI (blue) to visualize the nuclei and anti-GFP-Alexa Fluor-488 antibody (green) to visualize the EEFs. MSP-1 was stained with a mouse anti-MSP-1 primary antibody followed by an anti-mouse-Alexa Fluor555 secondary antibody (red). Scale bar = 20 µm. i) Merosome like structures from two independent experiments were injected IV into TO mice.

Supplementary Experimental Procedures:

Cell lines, parasite lines and mosquitoes

JM8.N4 and E14 mouse ESCs were maintained in Knockout™ Dulbecco's Modified Eagle Medium (DMEM, Gibco) and Glasgow's Minimum Essential Medium (GMEM, Sigma) respectively supplemented with fetal bovine serum (FBS), L-glutamine, β-mercaptoethanol (BME), non-essential amino acids (NEAA) and 1:10000 (1X) leukemia inhibitory factor (LIF, Millipore ESGRO 1107). Huh7 human hepatoma cells, NLSDM patient and healthy fibroblasts (received from Mojgan Reza, Newcastle Biobank, UK, after approval from the local ethics committee at Sanger Institute) were maintained in DMEM supplemented with FBS. iHLCs maintenance and differentiation method is described below. For all *P. berghei* sporozoite infection, the PbGFPcon parasite line previously derived from reference clone 15cy1 of the ANKA strain of *P. berghei* parasites was used (Franke-Fayard et al., 2004). Sporozoites were produced by feeding female *Anopheles stephensi* mosquitoes on Theiler's Origin (TO) mice infected with PbGFPcon parasite blood stocks intraperitoneally. All animal work was carried out under licenses from UK Home Office and protocols approved by the ethics committee at the Wellcome Trust Sanger Institute.

Sporozoite infection assay

P. berghei sporozoites isolated from *A. stephensi* salivary glands were added on top of cells in a ratio of 1:2 (parasites: cells) followed by centrifugation at 400 rcf, 4 ºC for 5 minutes. Infected cultures had a daily change of medium supplemented with 1% Pen/Strep (Gibco) and incubated at 37 ºC in 5 % CO_2.

MBA differentiation of mouse ESCs

A day before MBA treatment, E14 and JM8.N4 mouse ESCs were seeded on gelatin coated 24-well plates and cultured in their respective medium supplemented with 0.1X LIF (Millipore, ESGRO 1107). Next day, media containing 9 mM and 7 mM MBA (dissolved in DMSO) was added to E14 and JM8.N4 mouse ESCs respectively. MBA exposure lasted for 72 hours with fresh medium changes every day. After differentiation, MBA was removed and cells were cultured in their respective medium containing 0.1x LIF.

Nuclease assisted gene targeting in E14 mouse ESCs

Before nucleofection, E14 mouse ESCs were trypsinised and 2 x 10^6 cells were aspirated and centrifuged followed by removal of the supernatant. Next, 100 µl of Amaxa® Human Nucleofector® Kit 2 complete 'Solution 2' was added to the cells followed by transfer to the vial containing the prepared DNA (4 µg Cas9 expression plasmid, 2 µg targeting vector plasmid with 1 kb homology arms, 4µg guide RNA expression plasmid) and mixed 2-3 times. The cells-DNA mixture was quickly and gently transferred to the AMAXA nucleofection cuvette and electroporated immediately using the program'A-23'. Electroporated cells were plated on a gelatin coated Petri dish at various densities to ensure well-separated colonies for picking. Drug selection was applied 3 days post-nucleofection to allow time for transcription/translation of the Cas9 protein and guide RNAs and for homologous recombination to occur. To identify whether the targeted region is successfully disrupted, Long Range PCR (LRPCR) was performed using the LongAmp Taq Polymerase (New England BioLabs Cat# M0323). PCR products amplified from the non-targeted allele were Sanger sequenced.

Bulk mRNA sequencing

The Qiagen AllPrep DNA/RNA/miRNA Universal kit was used for isolating RNA from undifferentiated and MBA-differentiated E14 and JM8.N4 mESCs. A random-primed cDNA library was synthesized using Illumina's TruSeq Stranded mRNA Sample Prep Kit according. Next, cDNA libraries were amplified with KAPA HiFi Polymerase, quantified and pooled before sequencing on a HiSeq 2500 sequencer (total number of reads were 176,320,531, insert size ~150 bp, 92% reads mapped to *M. musculus* genome, read length 75bp PE, average fragment length 165bp, average yield per sample = 2895kb > Q20, Run 14037). R Graphical User Interface (v 3.1.3) was used for data analysis.

In vitro hepatocyte differentiation

Human iPSCs and ESCs were maintained in a chemically defined medium (CDM) containing polyvinyl alcohol (PVA, Sigma), bovine serum albumin (BSA), concentrated lipids, thioglycerols (Sigma-Aldrich), insulin (Roche), transferrin (Roche) and Pen/Strep. To begin differentiation, CDM-PVA containing basic fibroblast growth factor (bFGF) and Activin was added to iPSCs or ESCs on day 1. On day 2, definitive endoderm (DE) differentiation was induced by replacing medium with CDM-PVA containing Activin-A (100ng/mL), bFGF (100 ng/mL), BMP-4 (10 ng/mL), LY294002 (10 μM), and CHIR99021 (3 μM) for 24 hours. On Day 3, cells were fed with the same medium without CHIR99021. On day 4, CDM-PVA was replaced with RPMI-B27 medium (RPMI-1640, B27, NEAA and Pen/Strep) containing Activin-A (100 ng/mL), bFGF (100 ng/mL). Next, differentiation of anterior definitive endoderm was induced by incubating cells with RPMI-B27 medium containing Activin-A (50 ng/mL) from day 5 to 7 with media replacement every day. To induce hepatoblast specification, cells were treated with RPMI-B27 medium containing BMP-4 (20 ng/mL) and FGF10 (10 ng/mL) from day 8 to 12 with media replacement every day. From day 12 onwards, cells were allowed to functionally mature by feeding them with hepatocyte basal medium (Lonza CC-3199) containing OSM (30 ng/mL) and HGF (50 ng/mL). A change of medium was provided every alternate day during this phase of the protocol.

Western Blotting

Cell pellets were lysed in 1x LDS lysis buffer and boiled at 95°C for 10 minutes. Total protein concentration was measured through UV-protein estimation method and

increasing amount of protein (20 µg to 150 µg) was loaded on NuPAGE® Novex® 4-12% Bis-Tris Protein Gel in 1x running buffer and run at 200 V for 50 minutes. PageRuler™ Prestained Protein Ladder (10 to 180 kDa, Life Technologies (#26616)) was used for size determination. Proteins were transferred from NuPAGE® protein gel to nitrocellulose membrane (0.45 µ) at 30 V for 2 hours. Thereafter, the nitrocellulose membrane was blocked in 5% milk for 2 hours at room temperature (25°C) followed by overnight incubation with primary antibodies diluted (1:500) in blocking solution at 4°C with gentle shaking. All secondary antibodies were diluted (1:10000) in blocking solution and incubated with nitrocellulose membrane for 1.5 hours at room temperature (25°C) with gentle shaking. Proteins were detected on stained nitrocellulose membrane using the Amersham ECL Western Blotting Detection Reagent (GE Healthcare Life Sciences) according to the manufacturer's instructions.

Flow Cytometry

Flow cytometry samples were prepared by trypsinizing cells for 3 minutes at 37 ºC and stopping the reaction with appropriate ice-cold cell culture medium containing 10% FBS. A single cell suspension was achieved through repeated pipetting and centrifuged at 1100 rpm, 4 ºC for 3 minutes. The supernatant was aspirated and the cell pellet was washed with ice-cold 1% FBS solution in 1x PBS and centrifuged at 1100 rpm, 4ºC for 3 minutes. Finally, the pellet was re-suspended in 300 µl of 1% FBS solution and placed on ice. Infected cells were detected on the BD Fortessa by gating events on FSC vs SSC and GFP/Alexa 488 vs Alexa 647 dot plots. FlowJo (v 7.6.5) was used for data analysis. Please note, MBA differentiated mouse ESCs could not be recovered as intact single-cell suspension by enzymatic treatment and pipetting, thus, precluding infection quantification by flow cytometry based assay.

siRNA Reverse Transfection

Before transfection, 20 µM ATGL siRNA stock solution (Dharmacon) was diluted to a 2 µM ATGL siRNA working solution in RNase-free water. A pre-dilution of the siRNA was made in OptiMEM® (Gibco, cat#31985) by adding 1.5 µL of siRNA working solution to 8.5 µL of OptiMEM® and mixing gently. Next, a pre-dilution of Lipofectamine® RNAi MAX was made by gently mixing 0.2µL Lipofectamine® in 10 µL OptiMEM®. The siRNA pre-dilution and Lipofectamine® pre-dilution solutions

were mixed 1:1, mixed gently and incubated for 20 minutes at room temperature. Then, 20 µL of the siRNA/Lipofectamine® complexes was transferred to a new 96 well plate for cell culture. About 6000 cells were added per well in 80 µl final volume of RPMI 1640 supplemented with 10% FBS, 1% L-Glutamine, 1% NEAA and 1% HEPES. Edge wells were filled with water or PBS. The cells were incubated for 48h at 37ºC in 5% CO2 incubator.

Immunofluoroscence assay and microscopy

Cells were fixed with 4% PFA followed by permeabilization with 0.1% saponin. For blocking, cells were incubated with 1% BSA and 0.05% saponin solution in PBS for 1 hour. All primary antibodies (GFP-Alexa Fluor 488, Life Technologies; MSP-1, courtesy Anthony Holder at Francis Crick Institute, UK; HNF4a, SantaCruz sc6556; ATGL, Cell Signaling Technology #2138) were diluted in blocking solution and incubated with cells for 2 hours, followed by three washes with PBS. All secondary antibodies and the DNA staining dye DAPI (1:3000) were diluted in blocking solution and incubated with cells for 45 minutes in the dark. The High Content Screening (HCS) Thermo Scientific Cellomics imaging platform was used for image acquisition using the 'Acquire Only' algorithm. Cells were imaged at 200x magnification in DAPI and Alexa-488 channels with exposure times optimised beforehand. For parasite number and size quantification, 'Spot Detector' algorithm in HCS Studio Cellomics Scan software was used with object detection threshold determined before analysis. Late stage parasites stained with anti-MSP 1 antibody in iHLCs were imaged using Zeiss LSM 410 confocal microscope at 400x magnification.

Oil Red O staining

ORO powder (Sigma) was dissolved in 60 % isopropanol (in water) to prepare a 0.2 % solution and filtered through Whatman filter paper. Fixed cells were stained for 30 minutes with ORO solution and washed afterwards with 1X PBS.

Bibliography:

Franke-Fayard, B., Trueman, H., Ramesar, J., Mendoza, J., van der Keur, M., van der Linden, R., Sinden, R.E., Waters, A.P., and Janse, C.J. (2004). A Plasmodium berghei reference line that constitutively expresses GFP at a high level throughout the complete life cycle. Mol. Biochem. Parasitol. *137*, 23–33.
