# Supplementary material for: A Novel Chemically Differentiated Mouse Embryonic Stem Cell-Based Model to Study Liver Stages of Plasmodium berghei
Source: Stem Cell Reports. 2020 May 21;14(6):1123–34. doi: 10.1016/j.stemcr.2020.04.010 (PMC7355138; doi:10.1016/j.stemcr.2020.04.010)
Supplement: Document S2. Article plus Supplemental Information [file mmc4.pdf]

Stem Cell Reports
Article
OPEN ACCESS

A Novel Chemically Differentiated Mouse Embryonic Stem Cell-Based Model to Study Liver Stages of *Plasmodium berghei*

Jaishree Tripathi,[1] Charis-Patricia Segeritz,[2] Gareth Griffiths,[1] Wendy Bushell,[1] Ludovic Vallier,[1,2] William C. Skarnes,[3] Maria M. Mota,[4] and Oliver Billker[1,5,*]

[1]Wellcome Trust Sanger Institute, Wellcome Genome Campus, Hinxton, Cambridge, UK
[2]Wellcome Trust and Medical Research Council Stem Cell Institute, Department of Surgery, University of Cambridge, Cambridge, UK
[3]The Jackson Laboratory for Genomic Medicine, Ten Discovery Drive, Farmington, CT 06032, USA
[4]Unidade de Malária, Instituto de Medicina Molecular, Universidade de Lisboa, Lisboa, Portugal
[5]Molecular Infection Medicine Sweden and Molecular Biology Department, Umeå University, 90187 Umeå, Sweden
*Correspondence: oliver.billker@umu.se
https://doi.org/10.1016/j.stemcr.2020.04.010

SUMMARY

Asymptomatic and obligatory liver stage (LS) infection of *Plasmodium* parasites presents an attractive target for antimalarial vaccine and drug development. Lack of robust cellular models to study LS infection has hindered the discovery and validation of host genes essential for intrahepatic parasite development. Here, we present a chemically differentiated mouse embryonic stem cell (ESC)-based LS model, which supports complete development of *Plasmodium berghei* exoerythrocytic forms (EEFs) and can be used to define new host-parasite interactions. Using our model, we established that host *Pnpla2*, coding for adipose triglyceride lipase, is dispensable for *P. berghei* EEF development. In addition, we also evaluated *in-vitro*-differentiated human hepatocyte-like cells (iHLCs) to study LS of *P. berghei* and found it to be a sub-optimal infection model. Overall, our results present a new mouse ESC-based *P. berghei* LS infection model that can be utilized to study the impact of host genetic variation on parasite development.

INTRODUCTION

Malaria is a devastating mosquito-borne infectious disease which caused an estimated 405,000 deaths worldwide in 2018, with 93% of the cases occurring in Africa (World Health Organization, 2019). A cycle of human malaria infection begins when an infected *Anopheles* mosquito inoculates *Plasmodium* sporozoites into the skin of a vertebrate host (Amino et al., 2006), from where the parasites trickle into the circulation and migrate toward liver to invade hepatocytes and form exoerythrocytic forms (EEFs) enclosed within the parasitophorous vacuole. Targeting the liver stage (LS) of *Plasmodium* parasites with antimalarial drugs and vaccines is an attractive strategy to interrupt infection, as this is an obligatory and asymptomatic phase of infection that leads to the onset of symptomatic intra-erythrocytic schizogony. Furthermore, human malaria parasites, such as, *P. vivax* and *P. ovale*, produce dormant liver forms, or hypnozoites, which serve as a reservoir of infection (Wells et al., 2010).

Discovery of novel host-parasite interactions crucial for EEF development has been severely inhibited due to lack of robust and reproducible genetically tractable malaria LS cellular models. So far, mainly human and mouse liver cancer cell lines (Huh7, HepG2, Hepa1-6), hepatic cell lines (HC04), and primary hepatocytes have been utilized for this purpose (Prudêncio et al., 2011). More recently, micro-patterned human hepatocyte-murine embryonic fibroblasts co-cultures (MPCCs) and *in-vitro*-differentiated human hepatocyte-like cells (iHLCs) were described as well (March et al., 2013; Ng et al., 2015). Although cell lines are convenient to maintain and have a well-characterized genome and transcriptome, they are immortalized, and show dysregulated gene expression and abnormal signaling. For instance, hepatoma cells exhibit low abundance of drug metabolizing enzymes and transporters (Guo et al., 2011), altered glycogen synthase kinase 3β (Desbois-mouthon et al., 2002), and Toll-like receptor 3 signaling (Khvalevsky et al., 2007), thus, displaying phenotypes not observed in primary hepatocytes. Primary hepatocytes, instead, have limited availability, short lifespan, and lose their hepatic features rapidly *in vitro*. In addition, both, primary hepatocytes and hepatoma cell lines are acquired from a small group of donors thus representing limited genetic diversity within the population. Conversely, induced pluripotent stem cells (iPSCs), can be derived from diverse human subjects thus allowing generation of customized infection models and studying donor-specific infection phenotypes or responses.

Mouse embryonic stem cells (ESCs) and human iPSCs are normal cells with virtually unlimited proliferative abilities that are highly amenable to genetic manipulation (Sander and Joung, 2014; Shen et al., 2014) and share the capacity to differentiate into many different cell types (Sterneckert et al., 2014). In addition to self-renewal and pluripotency, large resources of stem cell lines are now available to explore mouse gene functions or investigate the impact of human genetic variation in the context of pathogen-host interactions (Elling et al., 2017; Kilpinen et al., 2017). These features of pluripotent stem cells (PSCs) has facilitated the

This is an open access article under the CC BY license (http://creativecommons.org/licenses/by/4.0/).

development of genetically tractable PSC-based infection models for several pathogens, such as, *Plasmodium* parasites, *Salmonella typhimirium*, and hepatitis C virus (Ng et al., 2015; Schwartz et al., 2012; Yeung et al., 2015; Yiangou et al., 2016). With respect to iHLCs, liver infection models for hepatitis C virus and *Plasmodium* spp. have been reported (Ng et al., 2015; Schwartz et al., 2012). For malaria specifically, iHLCs have been shown to support the development of various human and rodent *Plasmodium* spp. up to mature schizonts (Ng et al., 2015). In addition, erythrocytes derived from mouse ESCs have also been shown to be successfully infected with *P. berghei* blood stages (Yiangou et al., 2016). These developments indicate that usage of human and mouse PSCs in infectious disease research is opening up a new strategy to interrogate host-parasite interactions and can exploit existing resources, such as the Knockout Mouse Project repository. To investigate ways of leveraging this potential for research into the LS of malaria parasites, we explored both human and mouse PSC-based infection models to study *P. berghei* liver infection.

Primarily, in this study, we explored 3-methoxybenzamide (MBA)-differentiated mouse ESCs as a model to study *P. berghei* LS infection. MBA treatment of mouse ESCs presents a short, 3-day chemical differentiation method that produces large, terminally differentiated epithelial-like cells, as opposed to 25- to 35-day-long protocols for generating iHLCs. Because *P. berghei* sporozoites are highly promiscuous and can infect a variety of differentiated cell types, we hypothesized that MBA-differentiated mouse ESCs may be permissive to infection too, and could provide a new malaria LS infection model. Our results show that MBA-differentiated mouse ESCs support full development of *P. berghei* EEFs characterized by formation of large liver schizonts and release of infectious merosomes. We utilized this model to screen for host genes required for LS parasite development. In particular, we assessed the role of mouse *Pnpla2*, coding for adipose triglyceride lipase (ATGL), in *P. berghei* LS development in MBA-differentiated mouse ESCs, since preliminary small interfering RNA (siRNA) screening in Huh7 human hepatoma cells showed a negative impact on EEF development upon ATGL knockdown (see Results). In parallel, we re-examined the suitability of human PSC-derived iHLCs to study *P. berghei* LS infection *in vitro*. Our findings suggest that fully differentiated iHLCs (day 26 of differentiation) can be infected by *P. berghei* sporozoites but do not support complete development of EEFs, as depicted by the presence of small intrahepatic parasites several hours post-invasion, abnormal merozoite surface protein-1 (MSP-1) staining in liver schizonts, and lack of infectious merosomes. Overall, our results demonstrate a robust genetically modifiable mouse ESC-based *P. berghei* LS infection model which can be used to study novel and/or validate existing host-parasite interactions.

RESULTS

MBA-Differentiated Mouse ESCs Support Complete Development of *P. berghei* LS

MBA, is an inhibitor of ADP ribosyltransferase and is known to induce differentiation in mouse ESCs within 3 days of exposure (Smith, 1991). To assess the suitability of MBA-differentiated mouse ESCs as a model to study LS of *P. berghei*, we exposed two mouse ESC lines, JM8.N4 and E14, originally derived from C57Bl6/N (Pettitt et al., 2009) and 129P2/OlaHsd mice (Hooper et al., 1987), respectively, to MBA. Treatment of both cell lines with MBA for 72 h resulted in a monolayer of large, flat, terminally differentiated cells (Figures 1A and S1A). In our experience, JM8.N4 mouse ESCs show more uniform differentiation compared to E14 mouse ESCs, which still have a few colonies of undifferentiated cells left after MBA treatment.

Upon infection with freshly isolated GFP-expressing *P. berghei* sporozoites, a range of parasite sizes were observed in both MBA-differentiated and -undifferentiated JM8.N4 and E14 cells (Figures 1B and S1B). On average, MBA-differentiated JM8.N4 cells showed significantly larger EEFs at 48 h post-infection (hpi) and 64 hpi compared with EEFs in undifferentiated JM8.N4 mouse ESCs (Figure 1B). Similarly, MBA-differentiated E14 cells showed large EEFs at 48 hpi. However, due to the presence of several small EEFs in remaining undifferentiated colonies of cells, the average EEF size was not significantly larger than EEFs in control E14 undifferentiated cells (Figure S1B). Completion of LS development was marked by release of infectious merosomes from both MBA-differentiated JM8.N4 and E14 cells between 60 and 72 hpi (Figures 1C and S1C). Only the supernatant of MBA-differentiated mouse ESCs yielded blood stage infection when injected into mice (Figures 1D and S1D). Furthermore, immunostaining of EEFs in differentiated JM8.N4 and E14 cells with MSP-1 antibody showed MSP-1 expression around newly formed daughter nuclei in a distinct invaginating pattern (Figures 1E and S1E), thus indicating maturation of liver schizonts. Overall, our results demonstrate that treatment with MBA is a robust differentiation protocol which renders JM8.N4 and E14 mouse ESCs fully supportive of *P. berghei* LS development.

RNA Sequencing Reveals Transcriptional Profile of MBA-Differentiated Mouse ESCs

We determined the transcriptional changes induced within host cells after MBA treatment, through RNA sequencing (RNA-seq). A principal component analysis revealed no major batch effects between biological triplicates performed for each treatment, i.e., differentiated versus undifferentiated cells (Figure 2A). Differential gene expression analysis performed using Bioconductor

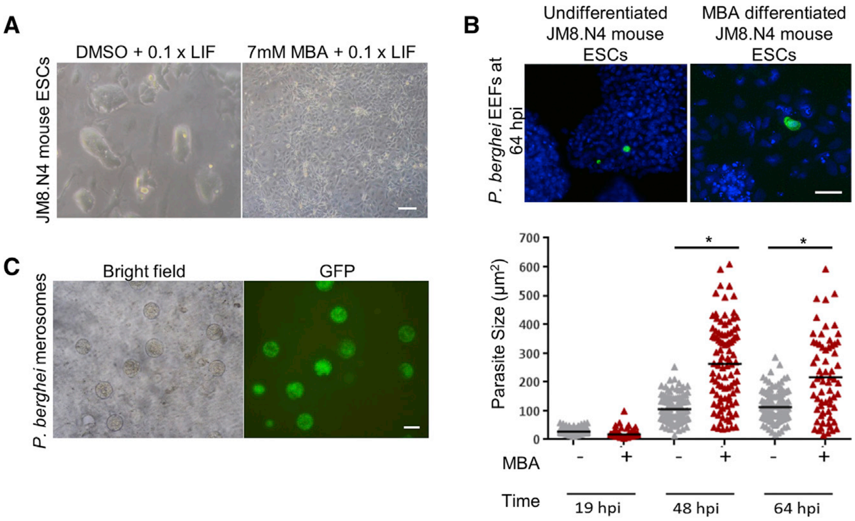

D

Cell Differentiation Status	Mice Injected with Merosomes/Supernatant	Mice Infected
MBA differentiated JM8.N4	5	5
Undifferentiated JM8.N4	5	0

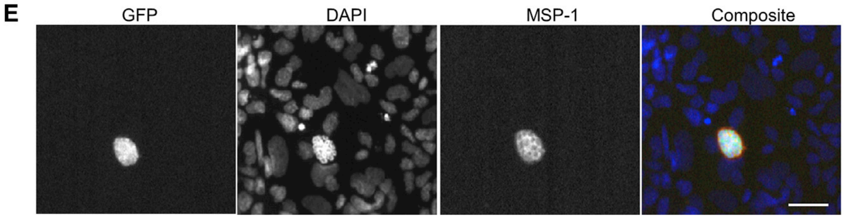

Figure 1. MBA-Differentiated JM8.N4 Mouse ESCs Support Complete *P. berghei* LS Development

(A) Morphology (bright-field image) of MBA- and DMSO (control)-treated JM8.N4 mouse ESCs on day 3 of differentiation.

(B) A fluorescence image panel of host cell nuclei and EEFs stained with DAPI (blue) and anti-GFP Alexa Fluor 488 antibody (green), respectively. A graph of EEFs sizes in DMSO- (gray) and MBA-treated (red) JM8.N4 mouse ESCs quantified through HTS Cellomics automated microscopy. Student's t test was performed on mean parasite size from three independent experiments. Asterisk (*) represents p value < 0.05.

(C) Bright-field and fluorescence image of merosomes released from infected MBA-treated JM8.N4 mouse ESCs beyond 60 hpi.

(D) Five Theiler's original (TO) mice were injected per condition with cell culture supernatant from infected, MBA- and DMSO-treated (undifferentiated) JM8.N4 cells at 72 hpi.

(E) MSP-1 expression in 65 hpi EEFs in MBA-differentiated JM8.N4 cells visualized by staining with mouse anti-MSP1 primary antibody and anti-mouse Alexa Fluor 555 secondary antibody (red) to visualize. DAPI (blue) and anti-GFP Alexa Fluor 488 antibody (green) staining shows nuclei and EEFs, respectively.

Scale bars, 250 µm (A) and 50 µm (B, C, and E).

package, DESeq, revealed 11,110 and 13,551 genes differentially expressed (DE) in MBA-differentiated JM8.N4 and E14 cells, respectively, with 9,366 DE genes common between the two cell lines. In particular, MBA-induced differentiation resulted in upregulation of smooth muscle-related genes, downregulation of pluripotency genes, and upregulation of host malaria LS-related genes in both JM8.N4 and E14 cells (Figure 2B). MBA differentiation coincided with a substantial downregulation of pluripotency markers, such as *Nanog*, *Rex2*, and *Oct3/4*. Loss of *Oct3/4* expression was confirmed through flow cytometry and microscopy in MBA-differentiated cells immunostained with anti-mouse Oct3/4 antibody (Figure S2). Furthermore, both MBA-differentiated JM8.N4 and E14 cells showed upregulation of several smooth muscle markers, such as smooth muscle actin 2 (*Acta2*), transgelin (*Tagln*), caldesmon (*Cald1*), and calponin 1 (*Cnn1*). A previous study has also reported increased expression of these genes in mouse ESC-derived contractile smooth muscle cells (Potta et al., 2009). Other genes, such as colony-stimulating factor 1 (*Csf-1*), filamin C gamma (*Flnc*), parvin alpha (*Parva*), cluster of differentiation 44 (*Cd44*), myosin 1C (*Myo1C*), claudin 6 (*Cldn6*), platelet/endothelial cell adhesion molecule 1 (*Pecam1*), smoothelin (*Smtn*), vinculin (*Vcl*), and calsyntenin 3 (*Clstn3*), known to be expressed in vascular smooth muscle cells (Sobue et al., 1999), were also found to be upregulated post-MBA differentiation. On comparison of transcriptome of MBA-differentiated mouse ESCs to transcriptomes of other cell types available on the MGI-Mouse Gene Expression Database (including smooth muscle cells) very low Pearson correlation values with maximum correlation (Pearson's r = 0.1) to trophoblast stem cell line R1AB (Table S1) was observed. Interestingly, a previous study has reported that trophoblast progenitor cells derived from the chorion express smooth muscle actin, vimentin, and β3 tubulin (Genbacev et al., 2011). In the context of LS infection, expression of typical hepatocyte markers such as albumin, tryptophan 2,3-dioxygenase, and liver fatty acid binding protein (*Lfabp*) was not identified in

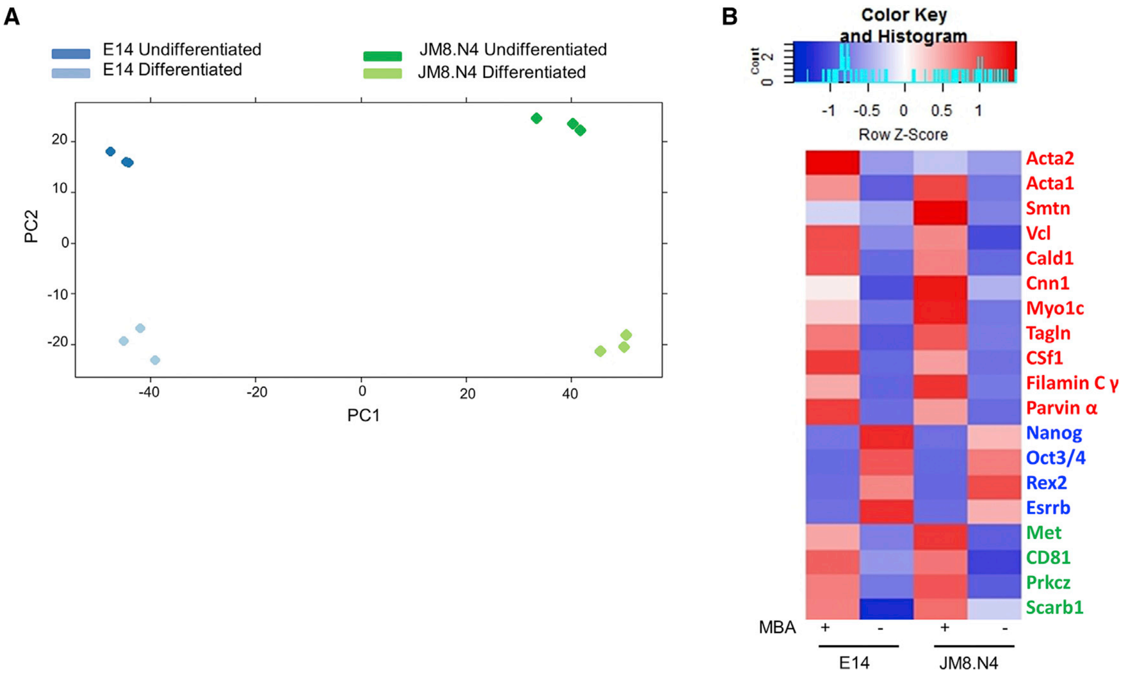

Figure 2. MBA-Differentiated Mouse ESCs Lose Expression of Pluripotency Markers and Upregulate Smooth Muscle Cell Markers

(A) A principal component analysis plot showing the first (PC1) and second principal component (PC2) separating the three biological replicates of undifferentiated and MBA-differentiated, JM8.N4 and E14 mouse ESCs, into separate clusters.

(B) A heatmap showing differentially expressed smooth muscle markers (red), pluripotency markers (blue), and genes previously implicated in malaria LS infection (green). Upregulation and downregulation are shown in red and blue, respectively.

MBA-differentiated JM8.N4 and E14 cells, confirming the lack of characteristic hepatocyte-like features. However, host factors previously implicated in malaria LS infection, such as cluster of differentiation 81 (*Cd81*), protein kinase C zeta (*Prkcz*), and hepatocyte growth factor receptor (*Hgfr*) (Carrolo et al., 2003; Prudêncio et al., 2011; Silvie et al., 2006) were upregulated in both JM8.N4 and E14 cells after MBA differentiation. Scavenger receptor class B type 1 (*Scarb1*), another host gene implicated in malaria LS infection (Yalaoui et al., 2008), was found to be upregulated only in MBA-differentiated E14 cells.

A gene ontology term enrichment analysis of genes DE in MBA-differentiated mouse ESCs (Table S2) revealed enrichment of lipid and amino acid metabolic processes, generation of precursor metabolites and energy, cytoskeleton organization, metal ion transport, Golgi vesicle transport, and pyrimidine ribonucleotide metabolic process among others. This indicates a highly metabolically active state of MBA-differentiated cells compared with their undifferentiated counterparts. Overall, our RNA-seq findings explain the conduciveness of MBA-differentiated mouse ESCs for development and maturation of *P. berghei* EEFs despite lacking typical hepatocyte features but probably sufficing the nutritional requirements of rapidly growing EEFs.

ATGL Is Dispensable for Development of *P. berghei* EEFs

Pnpla2, or patatin-like phospholipase domain-containing protein 2, codes for a 486-amino-acid-long protein called ATGL. ATGL hydrolyses triacylglycerols (TAGs) into diacylglycerol (DAG) and free fatty acid molecules in lipid storage organelles, such as lipid droplets (LDs). Previous studies have shown that the lipidome of *P. berghei* infected Huh7 human hepatoma cells changes significantly with a major alteration in neutral lipids, such as TAG, DAG, and cholesterol esters, suggesting that host lipogenesis and lipolysis pathway are engaged during *P. berghei* LS infection (Itoe et al., 2014). Interestingly, we found a decrease in the mean fluorescence intensity (MFI) of EEFs (measured by flow cytometry) in Huh7 cells transfected with *PNPLA2*-specific siRNA as compared with control transfected cells (Figure 3A). This suggests a negative impact on the development of intrahepatic parasites when host TAG hydrolysis and fatty acid mobilization is impaired in Huh7 hepatoma cells. On the contrary, *Pnpla2*-deficient mice show accumulation of LDs in hepatocytes, however, they are fully susceptible to *P. berghei* LS infection (Itoe et al., 2014). This discrepancy in results between *PNPLA2* gene knockdown *in vitro* and gene knockout in mice (*in vivo*) could be due to off-target effects of *PNPLA2* siRNA or the inaccuracy of

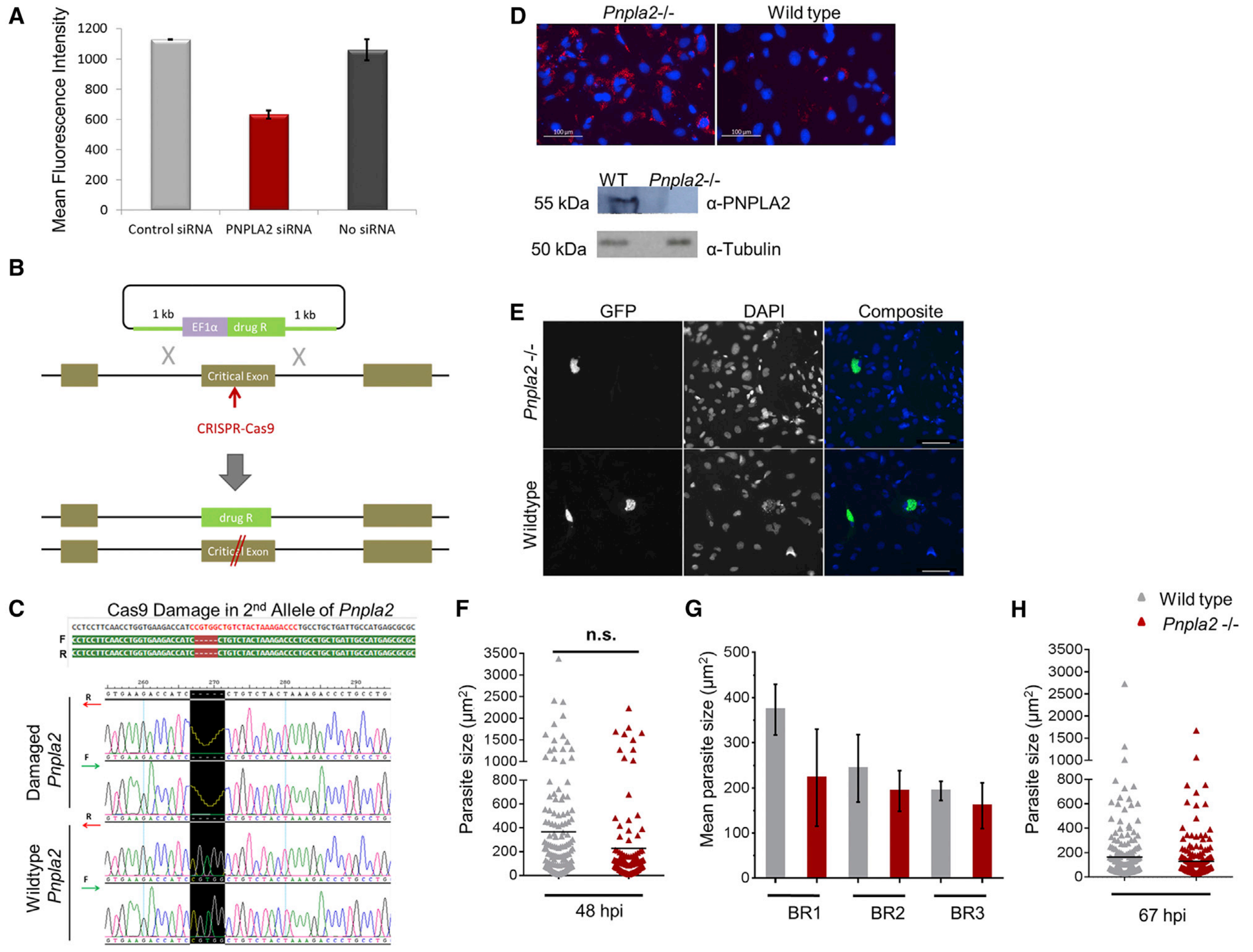

Figure 3. ATGL Is Dispensable for *P. berghei* EEF Development

(A) Huh7 human hepatoma cells were reverse transfected with control siRNA, *PNPLA2* siRNA, or transfection reagent (no siRNA). Twenty-four hours later 50,000 freshly isolated, GFP-expressing *P. berghei* sporozoites were added to each well and MFI was quantified at 48 hpi. Error bars represent standard deviation of the mean of two independent biological replicates.

(B) A schematic diagram showing biallelic targeting with CRISPR/Cas9 in mouse ESCs.

(C) *Pnpla2* "critical" exon was PCR amplified from genomic DNA of wild-type and *Pnpla2*$^{-/-}$ E14 mouse ESCs and sequenced. Cas9-damaged *Pnpla2* allele showed a 5-bp deletion compared with wild-type *Pnpla2* highlighted in black. F and R stand for forward and reverse strands, respectively.

(D) *Pnpla2*$^{-/-}$ and wild-type E14 cells fixed and stained with oil red O (ORO) and DAPI to visualize LDs and nuclei, respectively (scale bar, 100 μm). Western blot was performed on total cell lysate from *Pnpla2*$^{-/-}$ and wild-type E14 mouse ESCs.

(E) A widefield fluorescence image showing infected MBA-differentiated *Pnpla2*$^{-/-}$ and wild-type E14 mouse ESCs stained with DAPI (blue) and anti-GFP Alexa 488 antibody (green) to visualize host cell nuclei and EEFs respectively at 48 hpi. Scale bar, 100 μm.

(F) EEF size distribution quantified through HTS Cellomics automated microscopy is shown for a representative experiment (BR1) at 48 hpi. Student's t test was performed on mean parasite size from three independent biological replicates (n.s., not significant).

(G) A bar chart showing mean and standard deviation of parasite sizes at 48 hpi in all three biological replicates, BR1, BR2 and BR3.

(H) EEF size at 67 hpi quantified through HTS Cellomics automated microscopy. Mean represents average parasite size across triplicate wells from one experiment.

liver infection load assay in mice. Thus, we hypothesized that a more definitive analysis could be provided by *Pnpla2* knockout in our MBA-differentiated mouse ESCs model.

Pnpla2 knockout (*Pnpla2*$^{-/-}$) was generated in E14 mouse ESCs using a biallelic targeting strategy (Bressan et al., 2017) where sequence-specific CRISPR/Cas9 introduces a double-strand break in *Pnpla2* and deletion of the first allele occurs after integration of a drug resistance cassette from the donor vector through homologous recombination (Figure 3B). Damage in the second allele

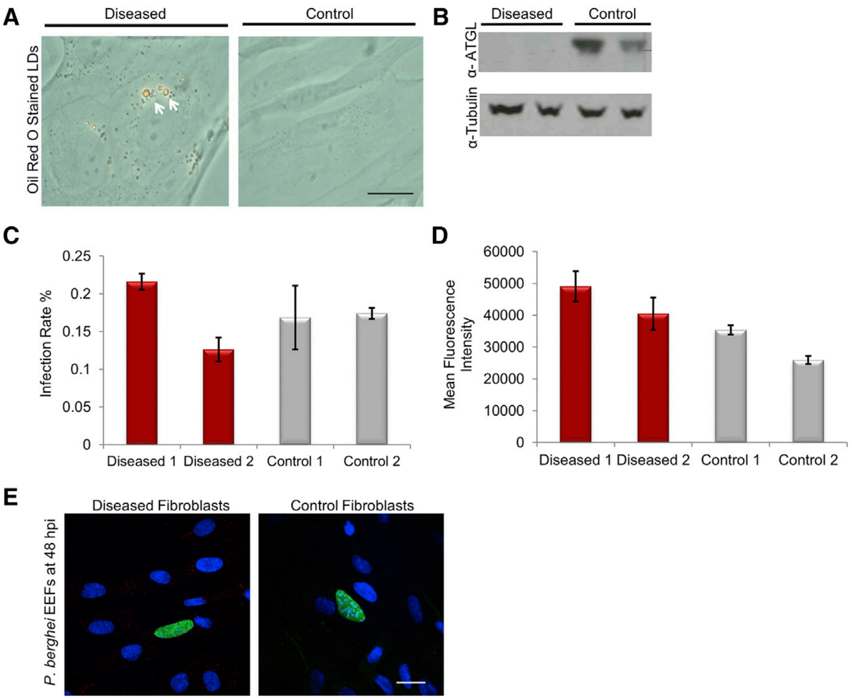

Figure 4. NLSDM Patient Fibroblasts with Point Mutations in *PNPLA2* Do Not Prevent *P. berghei* Infection and Growth

(A) A bright-field image of ORO staining of lipid droplets (white arrows) in healthy (control) and patient (diseased) fibroblasts. Scale bar, 2 μm.

(B) Western blot on total cell lysate from healthy and patient fibroblasts stained with rabbit anti-ATGL/PNPLA2 antibody (1:600) shows absence of PNPLA2 protein (~55 kDa) in patient fibroblasts. Anti-tubulin staining was performed as a loading control.

(C and D) (C) Infection rate and (D) MFI for *P. berghei*-infected diseased and control fibroblasts quantified by flow cytometry at 48 hpi. Error bars represent mean ± standard deviation of triplicates. Data shown are from a representative experiment out of two biological replicates.

(E) A representative confocal microscopy image showing *P. berghei* EEFs stained with anti-GFP Alexa Fluor 488 antibody in diseased and control fibroblasts at 48 hpi. Host cell nuclei were stained with DAPI. Scale bar, 20 μm.

is repaired through non-homologous end-joining which is an error-prone DNA repair mechanism often leading to frameshift mutations. *Pnpla2* gene disruption was confirmed in *Pnpla2*$^{-/-}$ E14 mouse ESCs through genotyping, which showed a 5-bp frameshift deletion in the "critical" exon (Figure 3C). Furthermore, MBA-differentiated *Pnpla2*$^{-/-}$ E14 cells showed substantial accumulation of large LDs in their cytosol as shown by oil red O staining of neutral triglycerides, and lacked ATGL protein compared with wild-type cells (Figure 3D). The enlargement and accumulation of LDs in mutant cells is thought to occur due to accumulation of TAGs in LDs after impaired interaction of ATGL with the LDs (Kobayashi et al., 2008).

After confirmation of *Pnpla2* disruption and loss of ATGL protein, MBA-differentiated *Pnpla2*$^{-/-}$ and wild-type E14 cells were infected with *P. berghei* sporozoites. At 48 hpi, the average parasite size in *Pnpla2*$^{-/-}$ E14 cells was slightly lower than in wild-type cells; however, this was not statistically significant (Figures 3E–3G). The total number of EEFs was also similar in both wild-type and mutated *Pnpla2* host genetic backgrounds (Figure S3). This suggests that sporozoite to EEF conversion is not affected in the absence of ATGL protein. To further check if the slight difference in EEF sizes observed at 48 hpi increased at later time points, parasite size was measured at 67 hpi and found to be similar in *Pnpla2*$^{-/-}$ and wild-type E14 cells (Figure 3H). These findings clearly indicate that *Pnpla2* is dispensable and is not required for *P. berghei* LS development in MBA-differentiated cells.

NLSDM Patient Fibroblasts Are Permissive to *P. berghei* Infection and Growth

Mutations in human *PNPLA2* gene causes neutral lipid storage disease with myopathy (NLSDM) characterized by severe accumulation of TAGs in cytoplasmic LDs in neutrophils, liver, muscle, and heart (Kobayashi et al., 2008; Schweiger et al., 2006). To test if natural genetic variation in *PNPLA2* affects susceptibility to malaria LS infection, we obtained two NLSDM patient dermal fibroblast lines F121-12N-YC280270 and F122-12N-LB141159 carrying mutation in exon 5 (613dupC) and exon 8 (1051delC), respectively (Reilich et al., 2011). Two healthy dermal fibroblast lines, F011-11N-RM01/02 and F097-12N-BH-01/02, were also received from the same source to be used as healthy controls in sporozoite infection assays. NLSDM patient fibroblasts showed accumulation of LDs in the cytosol and lacked ATGL protein when compared with healthy fibroblasts (Figures 4A and 4B). To check if LS phenotype observed in *Pnpla2*$^{-/-}$ E14 mouse ESCs could be reproduced in NLSDM fibroblasts, patient and healthy fibroblasts were infected with GFP-expressing *P. berghei* sporozoites in parallel. Both healthy and patient fibroblasts showed comparable infection rates at 48 hpi (Figure 4C). Furthermore, patient fibroblast line labeled "Diseased 1" showed slightly higher MFI than the two healthy control fibroblast

lines. However, the MFI of second patient fibroblast line labeled “Diseased 2” was comparable with healthy fibroblasts (Figure 4D). These results were validated through confocal microscopy where EEFs in patient and healthy fibroblasts looked comparable in size at 48 hpi (Figure 4E).

It is clearly evident from these results that both patient and healthy fibroblast lines show variation in infection rate and/or MFI value between themselves. This could be due to difference in their genetic background. To avoid being confounded by difference in host genetic backgrounds contributing to variation in infection and parasite development between fibroblast lines, *PNPLA2* knockdown was performed in healthy fibroblast line F011-11N-RM-01/02 (Control 1) and infected with *P. berghei* sporozoites to determine the role of *PNPLA2* during LS infection (Figure S4). *PNPLA2* transcript knockdown measured by qRT-PCR showed only 16% of transcript remaining in healthy fibroblasts at 48 h post-transfection (i.e., 84% transcript knockdown) compared with control siRNA transfected fibroblasts (Figure S4A). *P. berghei* infection rate and MFI in *PNPLA2* siRNA transfected fibroblasts was not significantly different from control transfected cells (Figures S4B and S4C). This confirms that the reduced EEF development observed in *PNPLA2* knockdown Huh7 hepatoma cells (Figure 3A) could not be reproduced, both, in MBA-differentiated *Pnpla2*$^{-/-}$ E14 mouse ESCs, and, NLSDM patient fibroblasts. Thus, PNPLA2 protein is dispensable for *P. berghei* LS infection in both, differentiated mouse ESCs, and, human fibroblasts. Overall, these results highlight the utility of MBA-differentiated mouse ESCs to validate the role of candidate host genes previously identified through gene knockdown approaches which may have frequent off-target hits leading to false positives.

iHLCs Do Not Support Complete Development and Maturation of *P. berghei* EEFs

The use of NLSDM fibroblasts above illustrates the potential of exploiting genetic variation in primary cells from different human donors to understand host-pathogen interactions. Dozens of candidate loci in the human genome have been linked to severe malaria in humans through genome wide association studies (Malaria Genomic Epidemiology Network et al., 2015; Timmann et al., 2012). Libraries of human iPSCs are now becoming available that reflect much of the most abundant variation in the human genome (Agu et al., 2015; Soares et al., 2014). We, therefore, tested if human ESC- or iPSC-derived hepatocyte-like cells can be used as a model to study *Plasmodium* LS infection. In this study, we utilized iHLCs derived through a 25- to 35-day-long differentiation of human ESCs or iPSCs into definitive endoderm cells, which gave rise to hepatic progenitors eventually maturing into hepatocytes (Hannan et al., 2013). iHLCs generated by this protocol have been characterized previously and display hepatic characteristics, such as expression of albumin, alpha fetal protein, glycogen storage, and cytochrome activity from day 20 onward. Basic hepatic metabolic functions start to appear from day 25 onward with increasing maturation up to day 35 (Touboul et al., 2010). The number of days required for hepatoblast maturation into iHLCs may vary between different cell lines and needs to be optimized in each case (Hannan et al., 2013).

In this study, we derived wild-type A1ATcorr iHLCs from A1ATcorr hiPSCs (Yusa et al., 2011) and observed for any morphological and phenotypic traits similar to primary hepatocytes. A1ATcorr iHLCs show a typical hepatocyte-like polygonal morphology with binucleate cells (Figure 5A) and express hepatocyte nuclear factor 4α (HNF4α) (Figure 5B), which is a transcription factor required for maintaining hepatic gene expression, bile acids secretion and lipid (e.g., triglycerides and cholesterol) homeostasis in hepatocytes (Hayhurst et al., 2001). HNF4α has been shown expressed in nascent hepatic cells, hepatoblasts and differentiated hepatocytes, thus marking liver-specific differentiation (Si-tayeb et al., 2010). Heterogeneous expression of HNF4α in the nuclei of A1ATcorr iHLCs reflects varied extent of differentiation of each cell. This is corroborated by previous studies which have shown that only 60% of iHLCs show HNF4 α expression and 80% show albumin expression which is another marker of hepatocyte differentiation (Si-tayeb et al., 2010; Touboul et al., 2010). No HNF4α expression was detected in undifferentiated A1ATcorr hiPSCs nuclei (negative control) (Figure 5B). Having confirmed the hepatocyte-like features of iHLCs, we systematically studied *P. berghei* infection, growth and maturation in them. As shown in Figure 5C, the infection rate (percentage of infected host cells) in A1ATcorr iHLCs infected with *P. berghei* sporozoites was about 0.21%, which was significantly lower compared with 0.53% in Huh7 human hepatoma cells (used as a comparator) at 48 hpi. Further development of *P. berghei* EEFs at 48 hpi was assessed by measuring parasite size using automated imaging. Although multiple parasite daughter nuclei were observed in both cell types, the average parasite size in A1ATcorr iHLCs was about 136.13 μm^2 compared with slightly higher 156.30 μm^2 in Huh7 cells (Figure 5D). A frequency distribution of EEF size in A1ATcorr iHLCs was similar to Huh7 cells except that fewer large EEFs between 300 and 600 μm^2 were present in iHLCs at 48 hpi (Figure 5E). Finally, the maturation of EEFs in A1ATcorr iHLCs was studied by staining infected cells with anti-MSP-1 antibody. MSP-1 is expressed in the plasma membrane of late liver schizonts and marks late LS maturation (Sturm et al., 2009). The pattern of MSP-1 staining in parasite plasma membrane indicates the extent of EEF maturation and membrane invagination around newly formed daughter

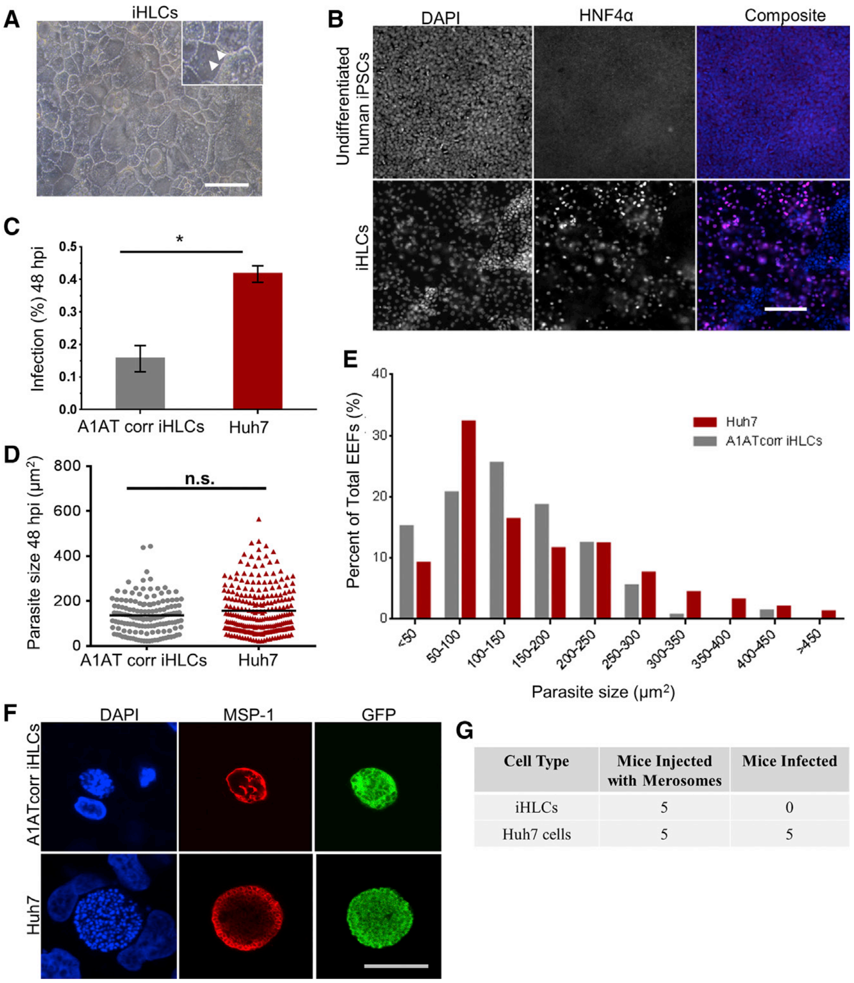

Figure 5. iHLCs Do Not Support Complete Development of *P. berghei* EEFs

(A) A bright-field image of iHLCs showing polygonal, binucleate (white arrows) hepatocyte-like morphology.

(B) HNF4α expression in iHLCs and undifferentiated hiPSCs visualized by staining with goat anti-HNF4α primary antibody and anti-goat Alexa Fluor 596 secondary antibody (purple). Host cells were stained with DAPI (blue). Images are representative of more than three independent differentiations.

(C) Infection rate in A1ATcorr iHLCs and Huh7 cells at 48 hpi quantified by flow cytometry. Data represent mean ± standard error of the mean of three independent experiments. Student's t test was performed on mean of technical replicates from three independent experiments. p value < 0.05 shown as asterisk (*).

(D) EEF size in A1ATcorr iHLCs and Huh7 cells at 48 hpi. Mann-Whitney test was applied to test for statistically significant difference. Data represented from triplicate wells of a representative experiment. n.s., not.

(E) A frequency distribution (as percentage) of EEF sizes in A1ATcorr iHLCs and Huh7 cells at 48 hpi.

(F) A representative confocal image of GFP-expressing EEFs in A1ATcorr iHLCs and Huh7 cells stained with anti-MSP-1 antibody (red) and DAPI (blue) at 65 hpi.

(G) Merosome-like structures released at 72 hpi from infected A1ATcorr iHLCs and Huh7 cells were injected intravenously into TO mice from two independent experiments. Combined data are shown from both experiments.

Scale bars, 100 µm (A and B) and 20 µm (F).

nuclei (Graewe et al., 2011). EEFs in A1ATcorr iHLCs showed abnormal pattern of MSP-1 staining at 65 hpi, lacking segregation of newly formed daughter nuclei into distinct units (Figure 5F). This is characteristic of delay in maturation of EEFs. In contrast, Huh7 EEFs showed a typical grape-like pattern of MSP-1 staining around individual daughter parasites (Figure 5F). Keeping infected cultures beyond 65 hpi (up to 96 hpi) resulted in occasional release of few merosome-like structures from A1ATcorr iHLCs but these did not produce blood stage infection in Theiler's original (TO) mice upon intravenous injection. On the contrary, merosomes released from infected Huh7 cells (control) always produced a positive blood stage infection in mice (Figure 5G). Overall, these results suggest that A1ATcorr iHLCs are susceptible to *P. berghei* infection but EEFs do not attain complete maturation in them. To rule out inappropriate host genetic background as a possible cause of stunted parasite growth, iHLCs derived from H9 human ESCs (Thomson et al., 1998) and BBHX8 human iPSCs (Rashid et al., 2010) were infected with *P. berghei* sporozoites. None of the two genetic backgrounds supported complete maturation of *P. berghei* EEFs as well (Figure S5). In conclusion, our results show that iHLCs are a suboptimal model to study LS of *P. berghei* parasites.

DISCUSSION

We have shown that differentiation with MBA dramatically increases permissiveness of JM8.N4 and E14 mouse ESCs to *P. berghei* sporozoite infection in contrast to undifferentiated cells. Transcriptional profiling post-MBA differentiation revealed increase in expression of several smooth

muscle-related genes but lack of typical hepatocyte markers. Nonetheless, complete development of *P. berghei* EEFs in this model may be attributable to the expression of *Scarb1*, *Met*, *Cd81*, *Hgfr*, and *Prkcz*; host genes previously implicated in malaria LS infection. In addition, significant enrichment of lipid, amino acids, and ribonucleotide metabolic processes along with vesical transport pathways may indicate nutritionally rich and metabolically active host cell environment post-MBA differentiation. We have clearly demonstrated the utility of this infection model to validate LS phenotypes previously associated with candidate host genes identified in gene knockdown studies. In particular, we established that host *Pnpla2* involved in neutral lipid mobilization is dispensable for *P. berghei* LS infection in MBA-differentiated mouse ESCs. We corroborate this finding by showing that there is no significant difference in *P. berghei* LS infection between NLSDM patient fibroblasts and healthy fibroblasts.

Plasmodium parasites can synthesize fatty acids using the type II fatty acid synthesis (FAS II) pathway. This may possibly explain why lack of *Pnpla2* which hydrolyses TAGs to release fatty acids and DAGs does not impact growth of *P. berghei* EEFs. Another possibility could be involvement of redundant genes/pathways which may become a substitute for ATGL in *Pnpla2* knockout cells. Discrepancy in infection phenotype between *PNPLA2* siRNA knockdown and *Pnpla2* gene knockout could be due to off-target effects of siRNAs leading to false positive hits thus requiring validation in a gene knockout system. The widely used CRISPR/Cas9 system can introduce precise mutations in any genomic loci with minimal off-target effects to mutate or correct gene/s of interest in diverse mammalian cell lines, including stem cells (Ding et al., 2013; Gaj et al., 2013). This outlines a major advantage of developing stem cell-based malaria LS infection model. Overall, our results are a proof-of-principle that MBA-differentiated mouse ESCs can be used for future gene-infection phenotype validation studies along with identification of new host-parasite interactions occurring during malaria LS infection.

Next, we tested if a hESC- or hiPSC-based malaria LS infection model can be developed as this would allow us to recapitulate human genetic variation occurring in primary cells of donors and study its effect on malaria LS infection. We showed that iHLCs derived from hiPSCs (A1AT-corr and BBHX8) or hESCs (H9) can be infected with *P. berghei* sporozoites, but do not support functional maturation of EEFs into segmented liver schizonts and infectious merosomes. This could be due to lack of one or more host factors in iHLCs known to affect growth and maturation of *Plasmodium* LS. Some of the host factors known to affect invasion and EEF development include, L-FABP which is possibly involved in delivering fatty acids to parasitophorous vacuole via interaction with PVM protein UIS3 (Labaied et al., 2007), SRB1 which plays a role in *Plasmodium* sporozoite entry and possibly supplies cholesterol required for EEF growth (Yalaoui et al., 2008), and host kinases PKCzeta whose knockdown leads to reduced parasite load *in vitro* and *in vivo* (Prudêncio et al., 2008). In addition, host metabolism, immunity, apoptosis and ER stress-related pathways have also been previously implicated in parasite growth and survival (Albuquerque et al., 2009). Furthermore, incomplete EEF development in iHLCs could be due to acquisition of anti-parasitic factors or immune-competency during maturation into hepatocyte-like cells. Previous studies have shown that cell-autonomous host defense mechanisms, such as interferon induced reactive oxygen and nitrogen species, immunity-related GTPases mediated disruption of parasitophorous vacuole, autophagy, and nutrient restriction, are major strategies used by a diverse range of primary cells to target intracellular protozoa such as *Leishmania*, *Toxoplasma*, *Plasmodium*, and *M. tuberculosis* (MacMicking, 2012, 2014). In context with *P. berghei* LS infection, Liehl et al. (2014) showed that *Plasmodium* RNA is recognized by pattern recognition receptor in infected hepatocytes which induces a type I interferon response responsible for recruiting liver leukocytes to eliminate intracellular EEFs. In contrast, undifferentiated mouse ESCs are known to lack strong immunological response when infected with bacterial pathogens such as *Salmonella* and *Shigella in vitro* (Yu et al., 2009). This could possibly mean that as ESCs differentiate into primary hepatocytes they gain host defense factors required for targeting intracellular pathogens. In fact, Ng et al. show that hepatoblasts are more permissive to *P. falciparum* infection than developmentally mature iHLCs.

While our study shows that iHLCs are a sub-optimal model to study LS infection, a previous study demonstrated that *P. falciparum*, *P. vivax*, *P. yoelii*, and *P. berghei* EEFs can mature in stem cell-derived hepatocytes (Ng et al., 2015). The discrepancy between our results could be due to use of different human PSC lines in the two studies, which may present variably suitable host genetic background to support infection and/or to form fully mature hepatocyte-like cells. Ng et al. reported formation of large, multinuclear, MSP-1 expressing liver schizonts in iHLCs, however, release of infectious merosomes from the host cell has not been addressed. In our opinion, one of the major challenges which remains to be tackled to develop successful stem cell-based LS models is to further improve hepatocyte differentiation protocols to generate fully mature, adult primary hepatocyte-like cells from diverse human stem cell lines instead of featuring fetal hepatocyte characteristics (Baxter et al., 2015). Molecules such as cyclic AMP and other small molecules have been shown to increase

maturation of iHLCs (Ogawa et al., 2013; Shan et al., 2013) and may facilitate development of better iHLC-based models in future. In addition, it is possible that essential nutrients such as D-glucose may have limited availability to EEFs in iHLCs. Previous research has shown that a minimum of 2,000 mg/L/day of D-glucose is essential for LS maturation (Itani et al., 2014). Thus, in future, efforts can be focused on optimizing iHLC media composition and culture conditions to promote complete LS maturation.

Overall, this study assessed the suitability of both, mouse and human PSC-based *P. berghei* LS infection models. iHLCs prove to be a sub-optimal infection model, however, MBA-differentiated mouse ESCs should enable the validation and discovery of new host-parasite interactions which are essential for *P. berghei* intrahepatic development and can be targeted to devise novel therapeutic interventions to treat malaria.

EXPERIMENTAL PROCEDURES

Resource Availability

The accession number for the RNA sequencing data reported in this paper is European Nucleotide Archive: ERP121187.

Cell Lines, Parasite Lines, and Sporozoite Infection Assay

JM8.N4, E14, Huh7, and fibroblasts were maintained in their respective media at 37°C in 5% CO_2. Human iPSCs and ESCs were maintained in a chemically defined medium and differentiated into iHLCs using a previously published protocol (Hannan et al., 2013). PbGFPcon parasite line was used for all sporozoite infections. Full details on media composition and infection assay are provided in Supplemental Experimental Procedures.

MBA Differentiation of Mouse ESCs

E14 and JM8.N4 mouse ESCs were differentiated using 9 mM and 7 mM MBA treatment, respectively, for 3 days. Full details are provided in Supplemental Experimental Procedures.

SUPPLEMENTAL INFORMATION

Supplemental Information can be found online at https://doi.org/10.1016/j.stemcr.2020.04.010.

AUTHOR CONTRIBUTIONS

J.T. and O.B. designed the study and wrote the manuscript. J.T. performed all experiments and data analyses. C.-P.S. generated iHLCs and provided human iPSCs and ESCs. G.G. performed mouse ESC genotyping. W.B. and W.C.S. provided wild-type and knockout mouse ESCs lines. O.B., M.M.M., and L.V. supervised the study.

ACKNOWLEDGMENTS

We extend our gratitude to Tom Metcalf, Mandy Sanders, and the Sanger Institute sequencing team for technical assistance. J.T. received an EVIMalaR PhD fellowship (European Union's Seventh Framework Program). This research was funded by grants from the Wellcome Trust (WT) and Medical Research Council (MRC). C.P.S. is funded through a Children's Liver Disease Foundation studentship. L.V. is funded by ERC Relieve IMDs, Cambridge University Hospitals National Institute for Health Research Biomedical Research Center, and the core support grant from the WT and MRC to the WT-MRC Cambridge Stem Cell Institute.

Received: June 24, 2019
Revised: April 26, 2020
Accepted: April 27, 2020
Published: May 21, 2020

REFERENCES

Agu, C.A., Soares, F.A.C., Alderton, A., Patel, M., Ansari, R., Patel, S., Forrest, S., Yang, F., Lineham, J., Vallier, L., et al. (2015). Successful generation of human induced pluripotent stem cell lines from blood samples held at room temperature for up to 48 hr. Stem Cell Reports *5*, 660–671.

Albuquerque, S.S., Carret, C., Grosso, A.R., Tarun, A.S., Peng, X., Kappe, S.H.I., Prudêncio, M., and Mota, M.M. (2009). Host cell transcriptional profiling during malaria liver stage infection reveals a coordinated and sequential set of biological events. BMC Genomics *10*, 270.

Amino, R., Thiberge, S., Shorte, S., Frischknecht, F., and Menard, R. (2006). Quantitative imaging of *Plasmodium* sporozoites in the mammalian host. C. R. Biol. *329*, 858–862.

Baxter, M., Withey, S., Harrison, S., Segeritz, C.-P., Zhang, F., Atkinson-Dell, R., Rowe, C., Gerrard, D.T., Sison-Young, R., Jenkins, R., et al. (2015). Phenotypic and functional analyses show stem cell-derived hepatocyte-like cells better mimic fetal rather than adult hepatocytes. J. Hepatol. *62*, 581–589.

Bressan, R.B., Dewari, P.S., Kalantzaki, M., Gangoso, E., Matjusaitis, M., Garcia-diaz, C., Blin, C., Grant, V., Bulstrode, H., Gogolok, S., et al. (2017). Efficient CRISPR/Cas9-assisted gene targeting enables rapid and precise genetic manipulation of mammalian neural stem cells. Development *144*, 635–648.

Carrolo, M., Giordano, S., Cabrita-Santos, L., Corso, S., Vigário, A.M., Silva, S., Leirião, P., Carapau, D., Armas-Portela, R., Comoglio, P.M., et al. (2003). Hepatocyte growth factor and its receptor are required for malaria infection. Nat. Med. *9*, 1363–1369.

Desbois-mouthon, C., Eggelpoel, M.B., Beurel, E., Boissan, M., Dello, R., Cadoret, I.A., and Capeau, J. (2002). Dysregulation of glycogen synthase kinase-3P signaling in hepatocellular carcinoma cells. Hepatology *36*, 1528–1536.

Ding, Q., Regan, S.N., Xia, Y., Oostrom, L.A., Cowan, C.A., and Musunuru, K. (2013). Enhanced efficiency of human pluripotent stem cell genome editing through replacing TALENs with CRISPRs. Cell Stem Cell *12*, 393–394.

Elling, U., Wimmer, R.A., Leibbrandt, A., Burkard, T., Michlits, G., Leopoldi, A., Micheler, T., Abdeen, D., Zhuk, S., Aspalter, I.M., et al. (2017). A reversible haploid mouse embryonic stem cell biobank resource for functional genomics. Nature *550*, 114–118.

Gaj, T., Gersbach, C.A., and Barbas, C.F. (2013). ZFN, TALEN, and CRISPR/Cas-based methods for genome engineering. Trends Biotechnol. *31*, 397–405.

Genbacev, O., Donne, M., Kapidzic, M., Gormley, M., Lamb, J., Gilmore, J., Larocque, N., Goldfien, G., Zdravkovic, T., McMaster, M.T., et al. (2011). Establishment of human trophoblast progenitor cell lines from the chorion. Stem Cells *29*, 1427–1436.

Graewe, S., Rankin, K.E., Lehmann, C., Deschermeier, C., Hecht, L., Froehlke, U., Stanway, R.R., and Heussler, V. (2011). Hostile takeover by *Plasmodium*: reorganization of parasite and host cell membranes during liver stage egress. PLoS Pathog. *7*, e1002224.

Guo, L., Dial, S., Shi, L., Branham, W., Liu, J., Fang, J., Green, B., Deng, H., Kaput, J., and Ning, B. (2011). Similarities and differences in the expression of drug-metabolizing enzymes between human hepatic cell lines and primary human hepatocytes. Drug Metab. Dispos. *39*, 528–538.

Hannan, N.R.F., Segeritz, C., Touboul, T., and Vallier, L. (2013). Production of hepatocyte like cells from human pluripotent stem cells. Nat. Protoc. *8*, 430–437.

Hayhurst, G.P., Lee, Y.-H., Gilles, L., Ward, J.M., and Gonzalez, F.J. (2001). Hepatocyte nuclear factor 4α (nuclear receptor 2A1) is essential for maintenance of hepatic gene expression and lipid homeostasis. Mol. Cell Biol *21*, 1393–1403.

Hooper, M., Hardy, K., Handyside, A., Hunter, S., and Monk, M. (1987). HPRT-deficient (Lesch-Nyhan) mouse embryos derived from germline colonization by cultured cells. Nature *326*, 292–295.

Itani, S., Motomi, T., and Ishino, T. (2014). D-Glucose concentration is the key factor facilitating liver stage maturation of *Plasmodium*. Parasitol. Int. *63*, 584–590.

Itoe, M., Sampaio, J.L., Cabal, G.G., and Real, E. (2014). Host cell phosphatidylcholine is a key mediator of malaria parasite survival during liver stage infection. Cell Host Microbe *10*, 778–786.

Khvalevsky, E., Rivkin, L., Rachmilewitz, J., Galun, E., and Giladi, H. (2007). TLR3 signaling in a hepatoma cell line is skewed towards apoptosis. J. Cell. Biochem. *100*, 1301–1312.

Kilpinen, H., Goncalves, A., Leha, A., Afzal, V., Alasoo, K., Ashford, S., Bala, S., Bensaddek, D., Casale, F.P., Culley, O.J., et al. (2017). Common genetic variation drives molecular heterogeneity in human iPSCs. Nature *546*, 370–375.

Kobayashi, K., Inoguchi, T., Maeda, Y., Nakashima, N., Kuwano, A., Eto, E., Ueno, N., Sasaki, S., Sawada, F., Fujii, M., et al. (2008). The lack of the C-terminal domain of adipose triglyceride lipase causes neutral lipid storage disease through impaired interactions with lipid droplets. J. Clin. Endocrinol. Metab. *93*, 2877–2884.

Labaied, M., Harupa, A., Dumpit, R.F., Coppens, I., Mikolajczak, S.A., and Kappe, S.H.I. (2007). *Plasmodium yoelii* sporozoites with simultaneous deletion of P52 and P36 are completely attenuated and confer sterile immunity against infection. Infect. Immun. *75*, 3758–3768.

Liehl, P., Zuzarte-Luís, V., Chan, J., Zillinger, T., Baptista, F., Carapau, D., Konert, M., Hanson, K.K., Carret, C., Lassnig, C., et al. (2014). Host-cell sensors for *Plasmodium* activate innate immunity against liver-stage infection. Nat. Med. *20*, 47–53.

MacMicking, J.D. (2012). Interferon-inducible effector mechanisms in cell-autonomous immunity. Nat. Rev. Immunol. *12*, 367–382.

MacMicking, J.D. (2014). Cell-autonomous effector mechanisms against *Mycobacterium tuberculosis*. Cold Spring Harb. Perspect. Med. *4*, 1–22.

Malaria Genomic Epidemiology Network, Band, G., Rockett, K.A., Spencer, C.C., and Kwiatkowski, D.P. (2015). A novel locus of resistance to severe malaria in a region of ancient balancing selection. Nature *526*, 253–257.

March, S., Ng, S., Velmurugan, S., Galstian, A., Shan, J., Logan, D.J., Carpenter, A.E., Thomas, D., Sim, B.K.L., Mota, M.M., et al. (2013). A microscale human liver platform that supports the hepatic stages of *Plasmodium falciparum* and *vivax*. Cell Host Microbe *14*, 104–115.

Ng, S., Schwartz, R.E., March, S., Galstian, A., Gural, N., Shan, J., Prabhu, M., Mota, M.M., and Bhatia, S.N. (2015). Human iPSC-derived hepatocyte-like cells support *Plasmodium* liver-stage infection in vitro. Stem Cell Reports *4*, 348–359.

Ogawa, S., Surapisitchat, J., Virtanen, C., Ogawa, M., Niapour, M., Sugamori, K.S., Wang, S., Tamblyn, L., Guillemette, C., Hoffmann, E., et al. (2013). Three-dimensional culture and cAMP signaling promote the maturation of human pluripotent stem cell-derived hepatocytes. Development *140*, 3285–3296.

Pettitt, S.J., Liang, Q., Rairdan, X.Y., Moran, J.L., Prosser, H.M., Beier, D.R., Lloyd, K.C., Bradley, A., and Skarnes, W.C. (2009). Agouti C57BL/6N embryonic stem cells for mouse genetic resources. Nat. Methods *6*, 493–495.

Potta, S.P., Liang, H., Pfannkuche, K., Winkler, J., Chen, S., Doss, M.X., Obernier, K., Kamisetti, N., Schulz, H., Hübner, N., et al. (2009). Functional characterization and transcriptome analysis of embryonic stem cell-derived contractile smooth muscle cells. Hypertension *53*, 196–204.

Prudêncio, M., Rodrigues, C.D., Hannus, M., Martin, C., Real, E., Gonçalves, L.A., Carret, C., Dorkin, R., Röhl, I., Jahn-Hoffmann, K., et al. (2008). Kinome-wide RNAi screen implicates at least 5 host hepatocyte kinases in *Plasmodium* sporozoite infection. PLoS Pathog. *4*, e1000201.

Prudêncio, M., Mota, M.M., and Mendes, A.M. (2011). A toolbox to study liver stage malaria. Trends Parasitol. *27*, 565–574.

Rashid, S.T., Corbineau, S., Hannan, N., Marciniak, S.J., Miranda, E., Alexander, G., Huang-doran, I., Griffin, J., Ahrlund-richter, L., Skepper, J., et al. (2010). Modeling inherited metabolic disorders of the liver using human induced pluripotent stem cells. J. Clin. Invest. *120*, 3127–3136.

Reilich, P., Horvath, R., Krause, S., Schramm, N., Turnbull, D.M., Trenell, M., Hollingsworth, K.G., Gorman, G.S., Hans, V.H., Reimann, J., et al. (2011). The phenotypic spectrum of neutral lipid storage myopathy due to mutations in the PNPLA2 gene. J. Neurol. *258*, 1987–1997.

Sander, J.D., and Joung, J.K. (2014). CRISPR-Cas systems for editing, regulating and targeting genomes. Nat. Biotechnol. *32*, 347–355.

Schwartz, R.E., Trehan, K., Andrus, L., Sheahan, T.P., Ploss, A., Duncan, S.A., Rice, C.M., and Bhatia, S.N. (2012). Modeling hepatitis C

virus infection using human induced pluripotent stem cells. Proc. Natl. Acad. Sci. U. S. A. *109*, 2544–2548.

Schweiger, M., Schreiber, R., Haemmerle, G., Lass, A., Fledelius, C., Jacobsen, P., Tornqvist, H., Zechner, R., and Zimmermann, R. (2006). Adipose triglyceride lipase and hormone-sensitive lipase are the major enzymes in adipose tissue triacylglycerol catabolism. J. Biol. Chem. *281*, 40236–40241.

Shan, J., Schwartz, R.E., Ross, N.T., Logan, D.J., Thomas, D., Duncan, S.A., North, T.E., Goessling, W., Carpenter, A.E., and Bhatia, S.N. (2013). Identification of small molecules for human hepatocyte expansion and iPS differentiation. Nat. Chem. Biol. *9*, 514–520.

Shen, B., Zhang, W., Zhang, J., Zhou, J., Wang, J., Chen, L., Wang, L., Hodgkins, A., Iyer, V., Huang, X., et al. (2014). Efficient genome modification by CRISPR-Cas9 nickase with minimal off-target effects. Nat. Methods *11*, 399–402.

Si-tayeb, K., Noto, F.K., Nagaoka, M., Li, J., Battle, M.A., Duris, C., North, P.E., Dalton, S., and Duncan, S.A. (2010). Highly efficient generation of human hepatocyte-like cells from induced pluripotent stem cells. Hepatology *51*, 297–305.

Silvie, O., Charrin, S., Billard, M., Franetich, J., Clark, K.L., Van Gemert, G., Sauerwein, R.W., Dautry, F., Boucheix, C., and Rubinstein, E. (2006). Cholesterol contributes to the organization of tetraspanin-enriched microdomains and to CD81-dependent infection by malaria sporozoites. J. Cell Sci. *119*, 1992–2002.

Smith, A.G. (1991). Culture and differentiation of embryonic stem cells. J. Tissue Cult. Methods *13*, 89–94.

Soares, F.A.C., Sheldon, M., Rao, M., Mummery, C., and Vallier, L. (2014). International coordination of large-scale human induced pluripotent stem cell initiatives: Wellcome Trust and ISSCR workshops white paper. Stem Cell Reports *3*, 931–939.

Sobue, K., Hayashi, K., and Nishida, W. (1999). Expressional regulation of smooth muscle cell-specific genes in association with phenotypic modulation. Mol. Cell. Biochem. *190*, 105–118.

Sterneckert, J.L., Reinhardt, P., and Schöler, H.R. (2014). Investigating human disease using stem cell models. Nat. Rev. Genet. *15*, 625–639.

Sturm, A., Graewe, S., Franke-fayard, B., Retzlaff, S., and Bolte, S. (2009). Alteration of the parasite plasma membrane and the parasitophorous vacuole membrane during exo-erythrocytic development of malaria parasites. Protist *160*, 51–63.

Thomson, J.A., Itskovitz-eldor, J., Shapiro, S.S., Waknitz, M.A., Swiergiel, J.J., Marshall, V.S., and Jones, J.M. (1998). Embryonic stem cell lines derived from human blastocysts. Science *282*, 1145–1148.

Timmann, C., Thye, T., Vens, M., Evans, J., May, J., Ehmen, C., Sievertsen, J., Muntau, B., Ruge, G., Loag, W., et al. (2012). Genome-wide association study indicates two novel resistance loci for severe malaria. Nature *489*, 443–446.

Touboul, T., Hannan, N.R.F., Corbineau, S., Martinez, A., Martinet, C., Branchereau, S., Mainot, S., Strick-Marchand, H., Pedersen, R., Di Santo, J., et al. (2010). Generation of functional hepatocytes from human embryonic stem cells under chemically defined conditions that recapitulate liver development. Hepatology *51*, 1754–1765.

Wells, T.N.C., Burrows, J.N., and Baird, J.K. (2010). Targeting the hypnozoite reservoir of *Plasmodium vivax*: the hidden obstacle to malaria elimination. Trends Parasitol. *26*, 145–151.

World Health Organization (2019). World Malaria Report 2019 (WHO).

Yalaoui, S., Huby, T., Franetich, J.-F., Gego, A., Rametti, A., Moreau, M., Collet, X., Siau, A., van Gemert, G.-J., Sauerwein, R.W., et al. (2008). Scavenger receptor BI boosts hepatocyte permissiveness to *Plasmodium* infection. Cell Host Microbe *4*, 283–292.

Yeung, A.T.Y., Hale, C., Xia, J., Tate, P.H., Goulding, D., Keane, J.A., Mukhopadhyay, S., Forrester, L., Billker, O., Skarnes, W.C., et al. (2015). Conditional-ready mouse embryonic stem cell derived macrophages enable the study of essential genes in macrophage function. Sci. Rep. *5*, 8908.

Yiangou, L., Montandon, R., Modrzynska, K., Rosen, B., Bushell, W., Hale, C., Billker, O., Rayner, J.C., and Pance, A. (2016). A stem cell strategy identifies glycophorin C as a major erythrocyte receptor for the rodent malaria parasite *Plasmodium berghei*. PLoS One *11*, e0158238.

Yu, J., Rossi, R., Hale, C., Goulding, D., and Dougan, G. (2009). Interaction of enteric bacterial pathogens with murine embryonic stem cells. Infect. Immun. *77*, 585–597.

Yusa, K., Rashid, S.T., Strick-Marchand, H., Varela, I., Liu, P.-Q., Paschon, D.E., Miranda, E., Ordóñez, A., Hannan, N.R.F., Rouhani, F.J., et al. (2011). Targeted gene correction of α1-antitrypsin deficiency in induced pluripotent stem cells. Nature *478*, 391–394.

Stem Cell Reports, Volume *14*

Supplemental Information

A Novel Chemically Differentiated Mouse Embryonic Stem Cell-Based Model to Study Liver Stages of *Plasmodium berghei*

Jaishree Tripathi, Charis-Patricia Segeritz, Gareth Griffiths, Wendy Bushell, Ludovic Vallier, William C. Skarnes, Maria M. Mota, and Oliver Billker

A novel chemically differentiated mouse embryonic stem cell based model to study liver stages of *Plasmodium berghei*

Jaishree Tripathi[1], Charis-Patricia Segeritz[2], Gareth Griffiths[1], Wendy Bushell[1], Ludovic Vallier[1,2], William C. Skarnes[3], Maria M. Mota[4], Oliver Billker[1, 5*]

[1] *Wellcome Trust Sanger Institute, Wellcome Genome Campus, Hinxton, Cambridge, UK*

[2] *Wellcome Trust and Medical Research Council Stem Cell Institute, Department of Surgery, University of Cambridge, Cambridge, UK*

[3] *The Jackson Laboratory for Genomic Medicine, Ten Discovery Drive, Farmington, CT 06032*

[4] *Unidade de Malária, Instituto de Medicina Molecular, Universidade de Lisboa, Lisboa, Portugal*

[5] *Molecular Infection Medicine Sweden and Molecular Biology Department, Umeå University, 90187 Umeå, Sweden*

**Correspondence: oliver.billker@umu.se*

Supplementary Figures:

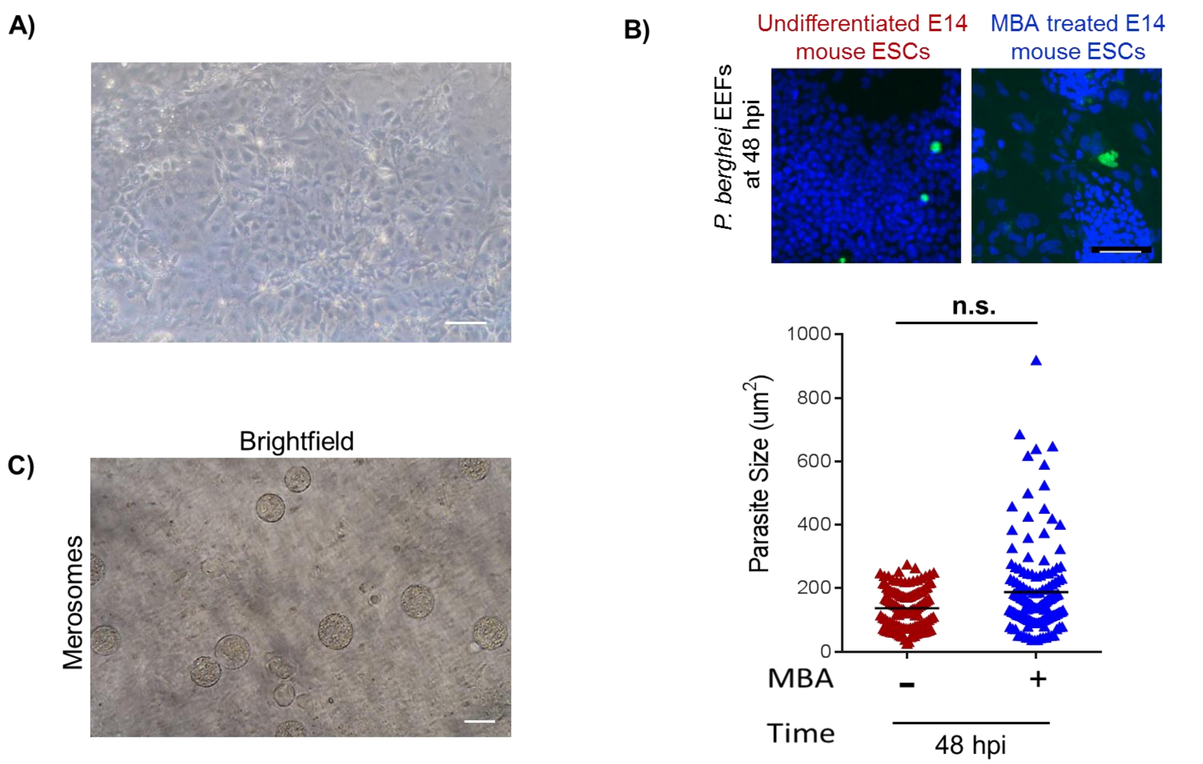

D)

Cell Type	Mice Injected with Merosomes/Supernatant	Mice Infected
MBA diff E14	5	5
Undiff E14	5	0

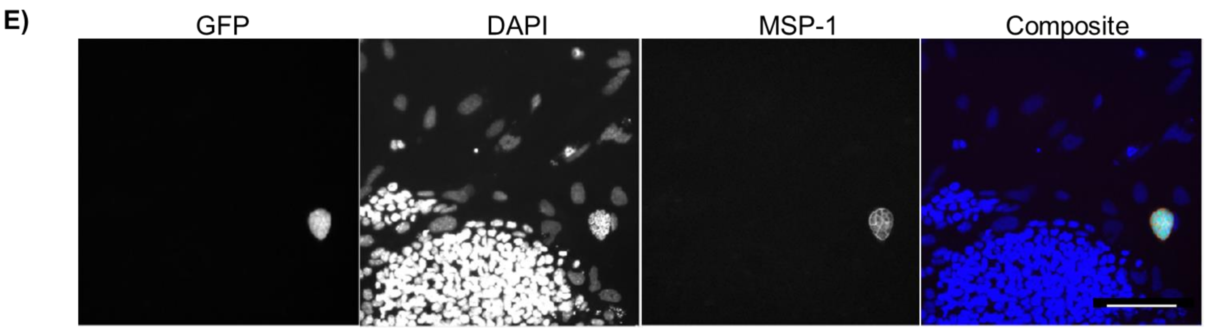

Fig S1: MBA-differentiated E14 mouse ESCs support complete *P. berghei* LS development. a) A bright field image showing morphology of MBA-treated E14 mouse ESCs on day 3 of differentiation. b) A widefield fluorescence image showing infected undifferentiated and MBA differentiated E14 mouse ESCs stained with DAPI (blue) and anti-GFP-Alexa-488 antibody (green) to visualize host cell nuclei and EEFs respectively at 48 hpi. Scale bar = 100 µm. Size of EEFs fixed at 48 hpi was quantified in undifferentiated (red) and MBA differentiated (blue) E14 mouse ESCs through automated

microscopy. Student’s t- test was performed on mean parasite size from three independent experiments. Key: n. s. = not significant. c) A bright field image of merosomes released from infected MBA differentiated E14 mouse ESCs. Scale bar = 50 µm. d) Two and three TO mice were injected per condition with cell culture supernatant from infected wells from two independent experiments respectively. e) Infected MBA differentiated E14 mouse ESCs were fixed, permeabilized and stained with mouse anti-MSP-1 primary antibody followed by anti-mouse-Alexa Fluor-555 secondary antibody (red) to visualize MSP-1 expression at 65 hpi. DAPI (blue) and anti-GFP-Alexa Fluor-488 antibody (green) staining was performed on the same culture to visualize nuclei and EEFs respectively. Scale bar = 100 µm.

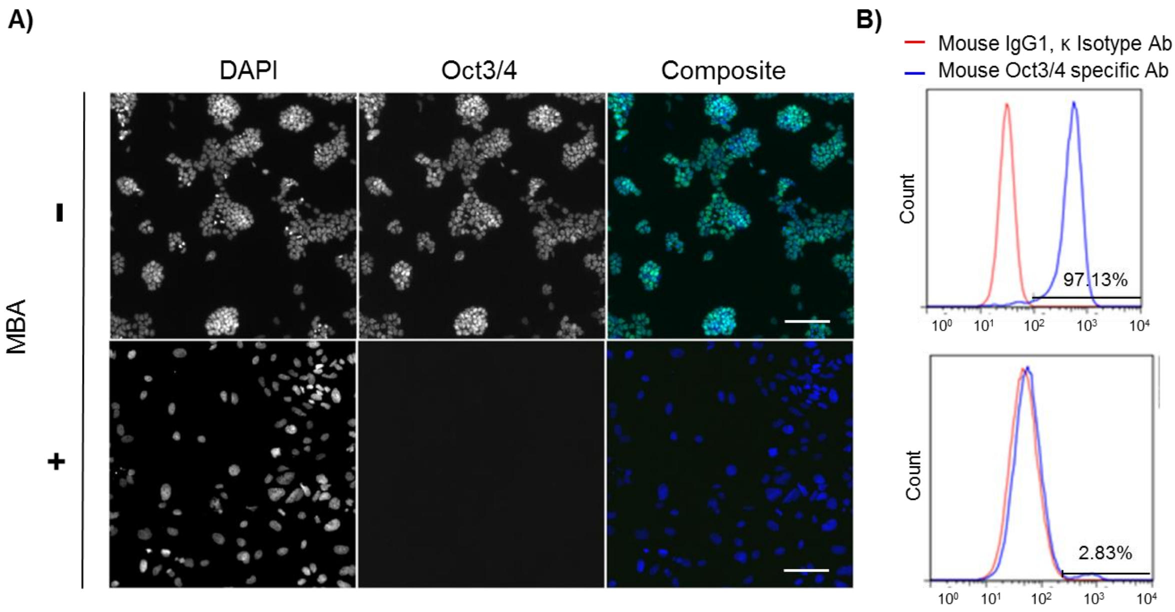

Fig S2: Loss of pluripotent marker expression after MBA treatment. Undifferentiated and MBA-differentiated E14 mouse ESCs were fixed, permeabilized and stained with a) DAPI and mouse anti-Oct3/4 primary antibody followed by donkey anti-mouse-Alexa 488 secondary antibody to visualise host cell nuclei and Oct3/4 transcription factors respectively. The imaging was done using wide field fluorescence microscopy. Scale bar = 100 µm. b) Mouse IgG1 isotype control (red) or mouse Oct3/4 specific antibody (blue) (BD Stemflow Human and Mouse Pluripotent Stem Cell Analysis Kit) to quantify undifferentiated (Oct3/4 positive) population by flow cytometry.

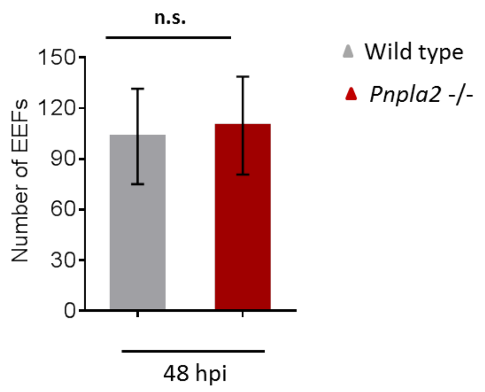

Fig S3: Number of EEFs in WT and Pnpla2 KO E14 cells. Number of EEFs in triplicate wells for three independent experiments. Error bar represents mean ± standard error of means (SEM). Key: n.s = not significant.

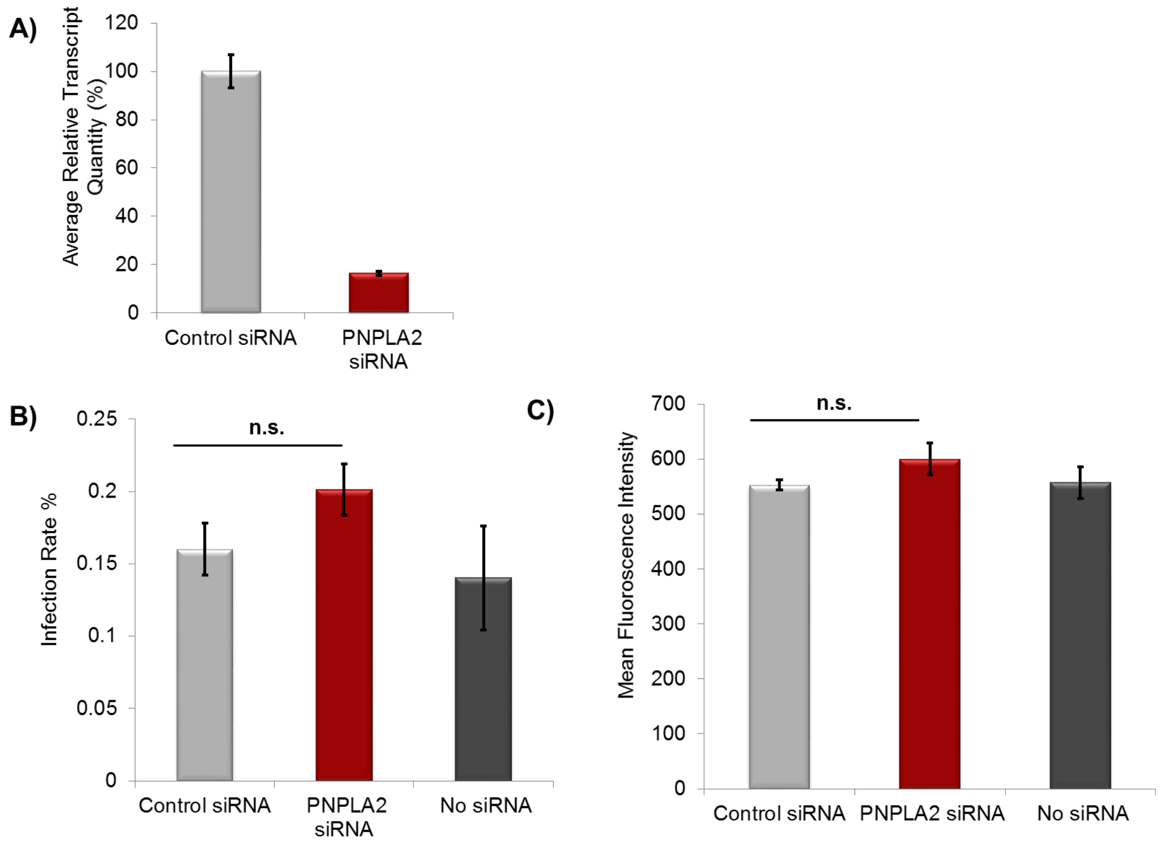

Fig S4: *PNPLA2* knockdown in healthy dermal fibroblast does not affect *P. berghei* infection and growth. a) *PNPLA2* transcript level was measured in control and *PNPLA2* siRNA transfected healthy fibroblasts by qRTPCR 48 hours after reverse transfection. Error bars represent mean ± standard deviation of duplicates from a representative experiment out of two independent biological replicates. b) Infection rate, and, c) MFI were measured in infected, control, *PNPLA2* and no siRNA transfected healthy fibroblasts at 48 hours post infection (hpi) by flow cytometry. Error bars represent mean ± standard deviation of triplicates from a representative experiment. Student's t test was

performed on mean infection rate (b) and MFI (c) from three independent experiments. n.s.= not significant.

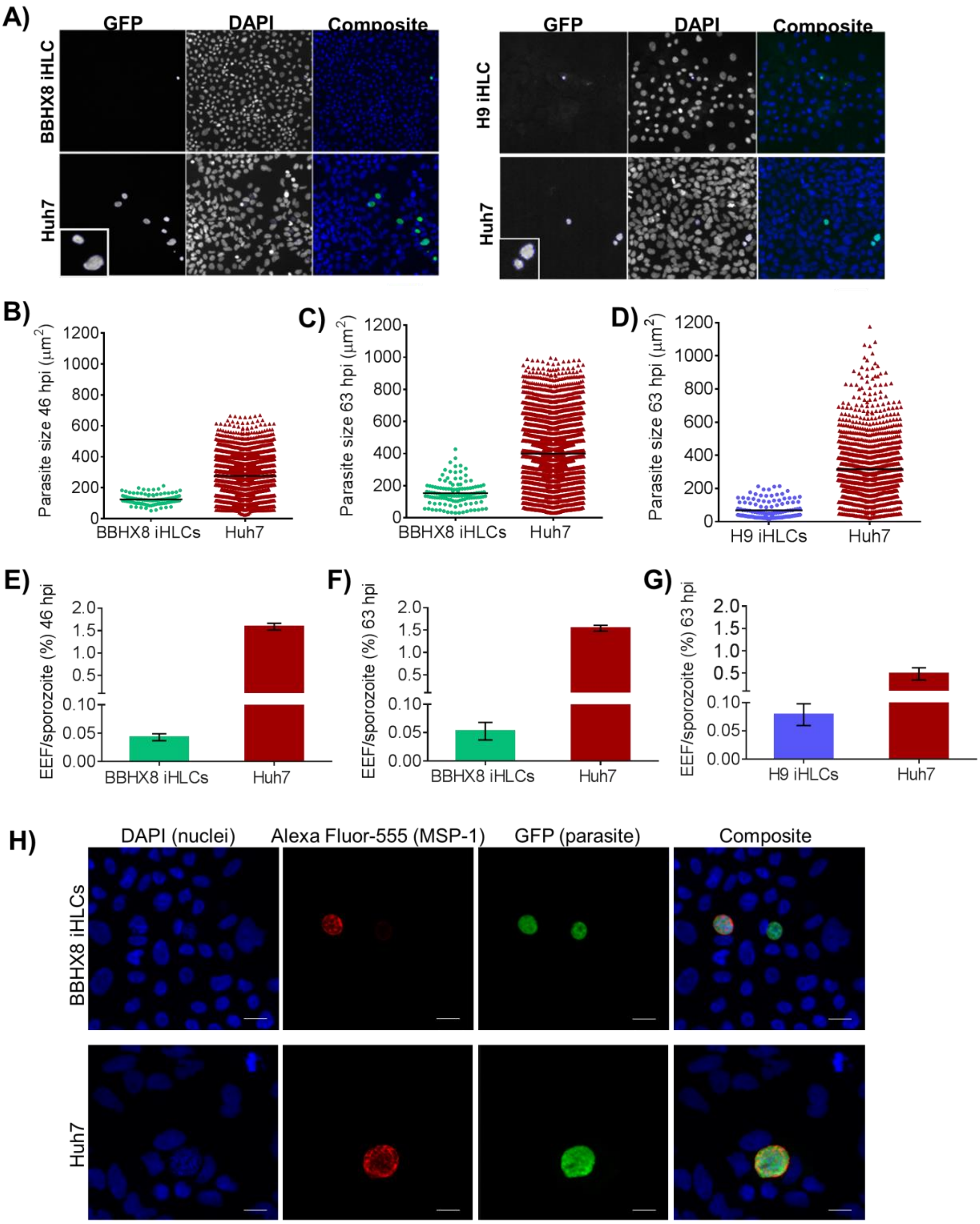

I)

Cell Type	Mice Injected with Merosomes	Mice Infected
BBHX8 iHLCs	4	0
Huh7 cells	4	4

Fig S5: *P. berghei* LS infection in BBHX8 and H9 iHLCs. a) Infected BBHX8 iHLCs, H9 iHLCs and Huh7 cells were fixed and stained with DAPI to visualize the nuclei (blue) and an anti-GFP-Alexa Fluor-488 antibody (green) to visualize the EEFs at 63 hpi. The EEFs were imaged automatically using the Spot Detector Algorithm. The objects detected by the algorithm are either accepted (denoted by blue ring) or rejected (denoted by orange ring) based on pre-set object selection parameters. b), c) and d) show parasite sizes in BBHX8 and H9 iHLCs at mid and late LS time points. Data shown here is from a representative experiment out of two independent biological replicates. e), f) and g) show sporozoite to EEF conversion (%) for BBHX8 and H9 iHLCs compared to their respective Huh7 controls. Data represents mean ± standard deviation of triplicates from a representative experiment out of two independent biological replicates. h) Infected BBHX8 iHLCs and Huh7 cells were fixed, permeabilized and stained with DAPI (blue) to visualize the nuclei and anti-GFP-Alexa Fluor-488 antibody (green) to visualize the EEFs. MSP-1 was stained with a mouse anti-MSP-1 primary antibody followed by an anti-mouse-Alexa Fluor555 secondary antibody (red). Scale bar = 20 µm. i) Merosome like structures from two independent experiments were injected IV into TO mice.

Supplementary Experimental Procedures:

Cell lines, parasite lines and mosquitoes

JM8.N4 and E14 mouse ESCs were maintained in Knockout™ Dulbecco's Modified Eagle Medium (DMEM, Gibco) and Glasgow's Minimum Essential Medium (GMEM, Sigma) respectively supplemented with fetal bovine serum (FBS), L-glutamine, β-mercaptoethanol (BME), non-essential amino acids (NEAA) and 1:10000 (1X) leukemia inhibitory factor (LIF, Millipore ESGRO 1107). Huh7 human hepatoma cells, NLSDM patient and healthy fibroblasts (received from Mojgan Reza, Newcastle Biobank, UK, after approval from the local ethics committee at Sanger Institute) were maintained in DMEM supplemented with FBS. iHLCs maintenance and differentiation method is described below. For all *P. berghei* sporozoite infection, the PbGFPcon parasite line previously derived from reference clone 15cy1 of the ANKA strain of *P. berghei* parasites was used (Franke-Fayard et al., 2004). Sporozoites were produced by feeding female *Anopheles stephensi* mosquitoes on Theiler's Origin (TO) mice infected with PbGFPcon parasite blood stocks intraperitoneally. All animal work was carried out under licenses from UK Home Office and protocols approved by the ethics committee at the Wellcome Trust Sanger Institute.

Sporozoite infection assay

P. berghei sporozoites isolated from *A. stephensi* salivary glands were added on top of cells in a ratio of 1:2 (parasites: cells) followed by centrifugation at 400 rcf, 4 ºC for 5 minutes. Infected cultures had a daily change of medium supplemented with 1% Pen/Strep (Gibco) and incubated at 37 ºC in 5 % CO_2.

MBA differentiation of mouse ESCs

A day before MBA treatment, E14 and JM8.N4 mouse ESCs were seeded on gelatin coated 24-well plates and cultured in their respective medium supplemented with 0.1X LIF (Millipore, ESGRO 1107). Next day, media containing 9 mM and 7 mM MBA (dissolved in DMSO) was added to E14 and JM8.N4 mouse ESCs respectively. MBA exposure lasted for 72 hours with fresh medium changes every day. After differentiation, MBA was removed and cells were cultured in their respective medium containing 0.1x LIF.

Nuclease assisted gene targeting in E14 mouse ESCs

Before nucleofection, E14 mouse ESCs were trypsinised and 2 x 10^6 cells were aspirated and centrifuged followed by removal of the supernatant. Next, 100 µl of Amaxa® Human Nucleofector® Kit 2 complete 'Solution 2' was added to the cells followed by transfer to the vial containing the prepared DNA (4 µg Cas9 expression plasmid, 2 µg targeting vector plasmid with 1 kb homology arms, 4µg guide RNA expression plasmid) and mixed 2-3 times. The cells-DNA mixture was quickly and gently transferred to the AMAXA nucleofection cuvette and electroporated immediately using the program'A-23'. Electroporated cells were plated on a gelatin coated Petri dish at various densities to ensure well-separated colonies for picking. Drug selection was applied 3 days post-nucleofection to allow time for transcription/translation of the Cas9 protein and guide RNAs and for homologous recombination to occur. To identify whether the targeted region is successfully disrupted, Long Range PCR (LRPCR) was performed using the LongAmp Taq Polymerase (New England BioLabs Cat# M0323). PCR products amplified from the non-targeted allele were Sanger sequenced.

Bulk mRNA sequencing

The Qiagen AllPrep DNA/RNA/miRNA Universal kit was used for isolating RNA from undifferentiated and MBA-differentiated E14 and JM8.N4 mESCs. A random-primed cDNA library was synthesized using Illumina's TruSeq Stranded mRNA Sample Prep Kit according. Next, cDNA libraries were amplified with KAPA HiFi Polymerase, quantified and pooled before sequencing on a HiSeq 2500 sequencer (total number of reads were 176,320,531, insert size ~150 bp, 92% reads mapped to *M. musculus* genome, read length 75bp PE, average fragment length 165bp, average yield per sample = 2895kb > Q20, Run 14037). R Graphical User Interface (v 3.1.3) was used for data analysis.

In vitro hepatocyte differentiation

Human iPSCs and ESCs were maintained in a chemically defined medium (CDM) containing polyvinyl alcohol (PVA, Sigma), bovine serum albumin (BSA), concentrated lipids, thioglycerols (Sigma-Aldrich), insulin (Roche), transferrin (Roche) and Pen/Strep. To begin differentiation, CDM-PVA containing basic fibroblast growth factor (bFGF) and Activin was added to iPSCs or ESCs on day 1. On day 2, definitive endoderm (DE) differentiation was induced by replacing medium with CDM-PVA containing Activin-A (100ng/mL), bFGF (100 ng/mL), BMP-4 (10 ng/mL), LY294002 (10 µM), and CHIR99021 (3 µM) for 24 hours. On Day 3, cells were fed with the same medium without CHIR99021. On day 4, CDM-PVA was replaced with RPMI-B27 medium (RPMI-1640, B27, NEAA and Pen/Strep) containing Activin-A (100 ng/mL), bFGF (100 ng/mL). Next, differentiation of anterior definitive endoderm was induced by incubating cells with RPMI-B27 medium containing Activin-A (50 ng/mL) from day 5 to 7 with media replacement every day. To induce hepatoblast specification, cells were treated with RPMI-B27 medium containing BMP-4 (20 ng/mL) and FGF10 (10 ng/mL) from day 8 to 12 with media replacement every day. From day 12 onwards, cells were allowed to functionally mature by feeding them with hepatocyte basal medium (Lonza CC-3199) containing OSM (30 ng/mL) and HGF (50 ng/mL). A change of medium was provided every alternate day during this phase of the protocol.

Western Blotting

Cell pellets were lysed in 1x LDS lysis buffer and boiled at 95°C for 10 minutes. Total protein concentration was measured through UV-protein estimation method and

increasing amount of protein (20 µg to 150 µg) was loaded on NuPAGE® Novex® 4-12% Bis-Tris Protein Gel in 1x running buffer and run at 200 V for 50 minutes. PageRuler™ Prestained Protein Ladder (10 to 180 kDa, Life Technologies (#26616)) was used for size determination. Proteins were transferred from NuPAGE® protein gel to nitrocellulose membrane (0.45 µ) at 30 V for 2 hours. Thereafter, the nitrocellulose membrane was blocked in 5% milk for 2 hours at room temperature (25°C) followed by overnight incubation with primary antibodies diluted (1:500) in blocking solution at 4°C with gentle shaking. All secondary antibodies were diluted (1:10000) in blocking solution and incubated with nitrocellulose membrane for 1.5 hours at room temperature (25°C) with gentle shaking. Proteins were detected on stained nitrocellulose membrane using the Amersham ECL Western Blotting Detection Reagent (GE Healthcare Life Sciences) according to the manufacturer's instructions.

Flow Cytometry

Flow cytometry samples were prepared by trypsinizing cells for 3 minutes at 37 ºC and stopping the reaction with appropriate ice-cold cell culture medium containing 10% FBS. A single cell suspension was achieved through repeated pipetting and centrifuged at 1100 rpm, 4 ºC for 3 minutes. The supernatant was aspirated and the cell pellet was washed with ice-cold 1% FBS solution in 1x PBS and centrifuged at 1100 rpm, 4ºC for 3 minutes. Finally, the pellet was re-suspended in 300 µl of 1% FBS solution and placed on ice. Infected cells were detected on the BD Fortessa by gating events on FSC vs SSC and GFP/Alexa 488 vs Alexa 647 dot plots. FlowJo (v 7.6.5) was used for data analysis. Please note, MBA differentiated mouse ESCs could not be recovered as intact single-cell suspension by enzymatic treatment and pipetting, thus, precluding infection quantification by flow cytometry based assay.

siRNA Reverse Transfection

Before transfection, 20 µM ATGL siRNA stock solution (Dharmacon) was diluted to a 2 µM ATGL siRNA working solution in RNase-free water. A pre-dilution of the siRNA was made in OptiMEM® (Gibco, cat#31985) by adding 1.5 µL of siRNA working solution to 8.5 µL of OptiMEM® and mixing gently. Next, a pre-dilution of Lipofectamine® RNAi MAX was made by gently mixing 0.2µL Lipofectamine® in 10 µL OptiMEM®. The siRNA pre-dilution and Lipofectamine® pre-dilution solutions

were mixed 1:1, mixed gently and incubated for 20 minutes at room temperature. Then, 20 µL of the siRNA/Lipofectamine® complexes was transferred to a new 96 well plate for cell culture. About 6000 cells were added per well in 80 µl final volume of RPMI 1640 supplemented with 10% FBS, 1% L-Glutamine, 1% NEAA and 1% HEPES. Edge wells were filled with water or PBS. The cells were incubated for 48h at 37ºC in 5% CO2 incubator.

Immunofluoroscence assay and microscopy

Cells were fixed with 4% PFA followed by permeabilization with 0.1% saponin. For blocking, cells were incubated with 1% BSA and 0.05% saponin solution in PBS for 1 hour. All primary antibodies (GFP-Alexa Fluor 488, Life Technologies; MSP-1, courtesy Anthony Holder at Francis Crick Institute, UK; HNF4a, SantaCruz sc6556; ATGL, Cell Signaling Technology #2138) were diluted in blocking solution and incubated with cells for 2 hours, followed by three washes with PBS. All secondary antibodies and the DNA staining dye DAPI (1:3000) were diluted in blocking solution and incubated with cells for 45 minutes in the dark. The High Content Screening (HCS) Thermo Scientific Cellomics imaging platform was used for image acquisition using the 'Acquire Only' algorithm. Cells were imaged at 200x magnification in DAPI and Alexa-488 channels with exposure times optimised beforehand. For parasite number and size quantification, 'Spot Detector' algorithm in HCS Studio Cellomics Scan software was used with object detection threshold determined before analysis. Late stage parasites stained with anti-MSP 1 antibody in iHLCs were imaged using Zeiss LSM 410 confocal microscope at 400x magnification.

Oil Red O staining

ORO powder (Sigma) was dissolved in 60 % isopropanol (in water) to prepare a 0.2 % solution and filtered through Whatman filter paper. Fixed cells were stained for 30 minutes with ORO solution and washed afterwards with 1X PBS.

Bibliography:

Franke-Fayard, B., Trueman, H., Ramesar, J., Mendoza, J., van der Keur, M., van der Linden, R., Sinden, R.E., Waters, A.P., and Janse, C.J. (2004). A Plasmodium berghei reference line that constitutively expresses GFP at a high level throughout the complete life cycle. Mol. Biochem. Parasitol. *137*, 23–33.
